# HLA Class I and II Blocks Are Associated to Susceptibility, Clinical Subtypes and Autoantibodies in Mexican Systemic Sclerosis (SSc) Patients

**DOI:** 10.1371/journal.pone.0126727

**Published:** 2015-05-20

**Authors:** Tatiana S. Rodriguez-Reyna, Pamela Mercado-Velázquez, Neng Yu, Sharon Alosco, Marina Ohashi, Tatiana Lebedeva, Alfredo Cruz-Lagunas, Carlos Núñez-Álvarez, Javier Cabiedes-Contreras, Gilberto Vargas-Alarcón, Julio Granados, Joaquin Zúñiga, Edmond Yunis

**Affiliations:** 1 Department of Immunology and Rheumatology, Instituto Nacional de Ciencias Médicas y Nutrición Salvador Zubirán, Mexico City, Mexico; 2 HLA laboratory, The American Red Cross Northeast Division, Dedham, Massachusetts, United States of America; 3 Department of Immunology, Instituto Nacional de Enfermedades Respiratorias Ismael Cosío Villegas, Mexico City, Mexico; 4 Basic and Technological Research Subdirection, Instituto Nacional de Cardiología Ignacio Chávez, Mexico City, Mexico; 5 Transplant Department, Instituto Nacional de Ciencias Médicas y Nutrición Salvador Zubirán, Mexico City, Mexico; 6 Department of Cancer Immunology and AIDS, Dana Farber Cancer Institute, Harvard Medical School, Boston, Massachusetts, United States of America; 7 Harvard Medical School, Boston, Massachusetts, United States of America; Medical University of South Carolina, UNITED STATES

## Abstract

**Introduction:**

Human leukocyte antigen (HLA) polymorphism studies in Systemic Sclerosis (SSc) have yielded variable results. These studies need to consider the genetic admixture of the studied population. Here we used our previously reported definition of genetic admixture of Mexicans using HLA class I and II DNA blocks to map genetic susceptibility to develop SSc and its complications.

**Methods:**

We included 159 patients from a cohort of Mexican Mestizo SSc patients. We performed clinical evaluation, obtained SSc-associated antibodies, and determined HLA class I and class II alleles using sequence-based, high-resolution techniques to evaluate the contribution of these genes to SSc susceptibility, their correlation with the clinical and autoantibody profile and the prevalence of Amerindian, Caucasian and African alleles, blocks and haplotypes in this population.

**Results:**

Our study revealed that class I block HLA-C*12:03-B*18:01 was important to map susceptibility to diffuse cutaneous (dc) SSc, HLA-C*07:01-B*08:01 block to map the susceptibility role of HLA-B*08:01 to develop SSc, and the C*07:02-B*39:05 and C*07:02-B*39:06 blocks to map the protective role of C*07:02 in SSc. We also confirmed previous associations of HLA-DRB1*11:04 and –DRB1*01 to susceptibility to develop SSc. Importantly, we mapped the protective role of DQB1*03:01 using three Amerindian blocks. We also found a significant association for the presence of anti-Topoisomerase I antibody with HLA-DQB1*04:02, present in an Amerindian block (DRB1*08:02-DQB1*04:02), and we found several alleles associated to internal organ damage. The admixture estimations revealed a lower proportion of the Amerindian genetic component among SSc patients.

**Conclusion:**

This is the first report of the diversity of HLA class I and II alleles and haplotypes Mexican patients with SSc. Our findings suggest that HLA class I and class II genes contribute to the protection and susceptibility to develop SSc and its different clinical presentations as well as different autoantibody profiles in Mexicans.

## Introduction

Systemic Sclerosis (SSc) is a connective tissue disorder characterized by inflammation, fibrosis, vasculopathy and autoimmune abnormalities, that affects several organs [1]. There are two main subsets of SSc according to the extent of the cutaneous involvement: diffuse cutaneous (dc) and limited cutaneous (lc) SSc. In the former, studies performed mainly in Caucasian and African American population, the most frequent autoantibodies are anti-topoisomerase I [2], anti-RNA polymerase I-III [3,4], and anti-U3 RNP [5]. Patients with lcSSc often express anticentromere [6] and anti Th/To antibodies [7]. Anti-U1 RNP, anti-PM-Scl and anti-Ku antibodies are usually present in SSc patients in overlap with other connective tissue disorders [8–11], and anti-U11/U12 RNP are present in SSc patients with severe interstitial lung disease [12].

The prevalence of these autoantibodies in Mexican SSc patients is different from that of other populations. Our patients have higher prevalence of anti-Topoisomerase I, anti-PM-Scl and anti-Ku antibodies and lower prevalence of anti-RNA polymerase III antibodies than other populations. Target organ damage associations with antibody presence remain the same than in other ethnic groups [13].

Genetic variations may contribute to differences in the clinical expression of the disease in several ethnic groups, and they could influence in the presence of SSc specific autoantibodies. Genome-wide studies have shown that the major histocompatibility complex (MHC) region plays a role in the susceptibility to develop SSc in Caucasians [14]. Genetic association studies in patients from different ethnic groups have shown that HLA class II genes are risk markers for SSc. Furthermore, HLA class II variability influences the autoantibody profile in SSc patients [15–20].

Since Systemic Sclerosis is a disease with variability in clinical presentation and in the prevalence of autoantibodies in different populations, it is of relevance to evaluate if the immunogenetic background of these patients influences their clinical and autoantibody profile.

In this study we determined HLA class I and II alleles using a high-resolution sequence-based typing method in a cohort of Mexican SSc patients. We evaluated their contribution to SSc susceptibility or protection in our population, their correlation with the clinical and autoantibody profile, and the prevalence of Amerindian, Caucasian and African alleles, haplotypes and blocks.

## Patients and Methods

### Patients

We included 159 patients from a cohort of SSc patients, without overlap with other autoimmune rheumatic diseases, and Mexican ancestry for at least 3 generations. All patients fulfilled ACR criteria for SSc [21] or LeRoy-Medsger criteria for early SSc [22]. Patients were evaluated by the rheumatologist responsible for the scleroderma cohort between July 2007 and July 2010 at the National Institute of Medical Sciences and Nutrition Salvador Zubirán, in Mexico City.

Patients were classified according to the clinical subset of the disease in diffuse cutaneous (dcSSc) and limited cutaneous systemic sclerosis (lcSSc) based on the extent of their skin involvement (skin involvement above elbows or knees or including chest or abdomen at any time during the illness for diffuse disease and distal to elbows or knees for limited disease). Organ involvement attributable to SSc was evaluated using previously published definitions, as follows: (a) peripheral vascular involvement was based on the presence of any of the following: Raynaud phenomenon, digital pitting scars, digital tip ulcerations, or digital gangrene; (b) joints/tendons involvement was defined as any of the following: arthritis, carpal tunnel syndrome, tendon friction rubs, or finger joint contractures (finger to palm distance in full flexion > 1 cm); (c) skeletal muscle involvement required the presence of proximal muscle weakness on physical examination and any of the following: elevated serum creatine kinase, myopathic changes on electromyography, or evidence of myositis on muscle biopsy; (d) gastrointestinal tract involvement required the presence of any of these manifestations: distal esophageal hypomotility or stricture by esophagogram, manometry or endoscopy, small bowel hypomotility by small intestine follow through, wide-mouth colonic sacculations, or malabsorption syndrome; (e) interstitial lung disease (ILD) was defined as forced vital capacity (FVC) < 70% of predicted, and forced expiratory volume in 1 s (FEV_1_)/FVC >80%, or interstitial fibrosis or ground glass changes on chest radiography or high-resolution computed tomography scan of the lungs; pulmonary arterial hypertension (PAH) was defined as mean PA pressure >25 mmHg by right heart catheterization or PA systolic pressure > 40 mmHg by echocardiogram; (f) cardiac involvement was defined as left-sided congestive heart failure (FEVI <45%) or pericarditis by echocardiogram or cardiac magnetic resonance imaging, arrhythmia requiring treatment, or conduction defect; and (g) renal involvement was recorded as history or presence of scleroderma renal crisis. The percentage of the patients with involvement of each organ system, was determined by the number of patients with documented organ involvement divided by the number of patients who had undergone objective evaluation of that organ. Severity of the disease was determined according to the Medsger severity scale [13, 23].

As a control group we included 234 healthy Mexican admixed individuals that donated a venous blood sample to determine HLA genes. All subjects came from the central area of Mexico and had Mexican ancestry for at least 3 generations. Mean age of studied individuals was 38 ± 15.3 years. There were 120 females (51%) and 114 males (49%).

### Ethics Statement

The Institutional Review Boards of the Instituto Nacional de Ciencias Médicas y Nutrición Salvador Zubiran (INCMNSZ) and the Instituto Nacional de Enfermedades Respiratorias Ismael Cosío Villegas (INER) approved the study. All subjects provided written informed consent for clinical data collection; serum and DNA sample collection and storage. Only adults older than 17 years were included. This study was carried out in accordance to the Helsinki Declaration contents.

### SSc specific autoantibodies

Peripheral venous blood with EDTA was obtained to test for SSc-associated antibodies (10 ml of blood to isolate serum that was frozen at -70°C until processed). Antinuclear antibodies (ANA) and specific autoantibodies were determined as previously described [13]. Briefly, antinuclear IgG antibodies were detected by indirect immunofluorescence using Hep-2 cell line as substrate, according to the manufacturer´s recommendations (The Binding Site, Birmingham, UK). Serum samples were tested at 1:40 dilution. Three experts read all the samples and results were discussed and registered. IgG isotype anti-topoisomerase-I, anticentromere B and anti-U1 RNP were detected by immunoenzymatic assay (EIA) according to the manufacturer´s recommendations (The Binding Site); IgG isotype anti-RNA Pol III antibodies were detected by EIA according to the manufacturer´s recommendations (INOVA Diagnostics, San Diego, CA, USA). Serum samples were diluted 1:100 and processed in an automated DSX System (DYNEX Technologies; Chantilly, VA, USA). The sera cut-off values of each antibody were set above the 90 percentile of a separate control group of 100 healthy individuals. For anticentromere A, anti-U3 RNP, anti-U11/U12 RNP, anti-PM-Scl, anti-Th/To, and anti-Ku antibodies, a commercial Western blot was performed using Hep-2 whole cell extract and recombinant, centromere A, U3 RNP, U11/U12 RNP, PM-Scl, Th/To, and Ku proteins according to the manufacturer´s recommendations (Euroimmun, Lübeck, Germany); briefly, serum samples (1:51) were incubated with the antigen for 1h at room temperature under shaking; after two washes, alkaline phosphatase-labeled goat anti-human IgG antibody was used to detect bound antibodies [13].

### HLA sequence based typing

Genomic DNA was isolated from peripheral blood mononuclear cells (PBMC), using the QIAamp DNA mini kit (Qiagen, Valencia, CA, USA). High-resolution HLA class I and class II typing was performed by a sequence-based typing method (SBT) as previously described [24–26]. Briefly, we amplified exon 2 and 3 from HLA-A, -B and –C and exon 2 for HLA-DRB1 and -DQB1. Polymerase chain reaction (PCR) contained 1.5 mM KCl, 1.5 mM MgCl_2_, 10 mM Tris-HCl (pH = 8.3), 200 mM concentrations of each dATP, dTTP, dGTP, and dCTP; 10 pM concentration of each primer, 30 ng of DNA and 0.5 U of Taq DNA polymerase in a final volume of 25 μl. Amplification was done on a PE9700 thermal cycler (Applied Biosystems, Foster City, CA, USA) using the following cycling conditions: 95°C for 30 s, 65°C for 30 s, 72°C for 1 min, preceded by 5 min at 95°C, and followed by a final elongation at 72°C for 5 min. Amplified products were sequenced independently in both directions using BigDye Terminator chemistry in an ABI PRISM 3730xl Genetic Analyzer (Applied Biosystems, Foster City, CA, USA) using the IMGT/HLA sequence database alignment tool (http://www.ebi.ac.uk/imgt/hla/align.html). Ambiguities were solved using group-specific sequencing primers (GSSPs) that have been reported and validated previously [26].

### HLA blocks and haplotypes assignment and admixture estimations

Conserved extended haplotypes (CEH’s) were estimated by maximum likelihood using the computer program Arlequin version 2.0 [26, 27].

Listed HLA-C-B, HLA-DRB1-DQB1 and CEHs of Amerindian origin were estimated by the maximum likelihood method based on the delta (D) and delta max (D') between alleles of two loci and between the two blocks and/or the extension to the HLA-A region, as previously described [26]. CEHs or DNA blocks of African, Asian and Caucasian most probably ancestry (MPA) were assigned based on previous reported frequencies [28–30]. Estimation of D and D' values to measure (linkage disequilibrium) LD, nonrandom association of alleles at two or more loci, and their statistical significance, were calculated using previously described methods [26, 30]. Absolute D' values of 1 indicates complete LD; 0 corresponds to no LD.

As an approach to estimate the diversity and contribution of previously described [28–30] Caucasian, Asian, and African HLA blocks in our population, we also calculated the aggregate block frequencies (ABF) as previously described and validated [26, 29, 30] adding the frequencies of those HLA class I and II blocks with frequencies greater than 1% in our study population.

Admixture estimates were obtained using the Leadmix software [31], with k = 3 parental populations (Native American, African and European) and HLA-B as the genetic estimator. Caucasian component was estimated using data from southern Portugal [32] and from an European population sample from USA [28]; Africans from Kenia [33] as the African parental component and a pooled Native American sample from Mixtec of Oaxaca, SE Mexico [34] and Tarahumara Mexican Amerindians [35].

**Fig 1:**
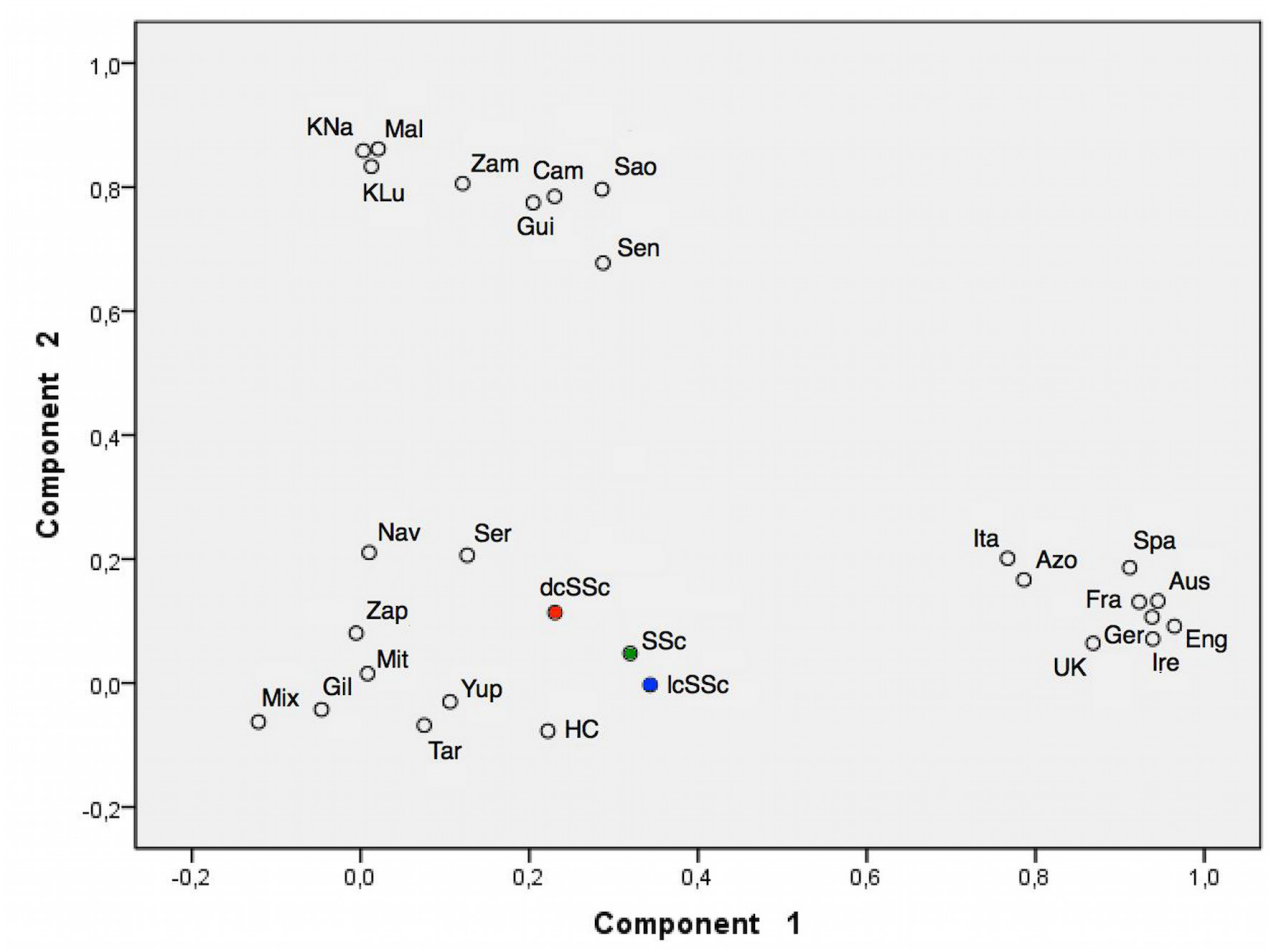
Principal component analysis (PCA) plot reveals a close genetic relationship of Mexican admixed SSc patients and healthy controls (HC) from Mexico City to Native American groups. Native American populations are represented in the upper left of the graphic and Caucasian components in the right bottom area of the graphic. Amerindian components are represented in the left bottom area. Red and blue dots represent difusse and limited SSc patients respectively and the total group in represented in green. The different populations included in the PCA analysis were: Ire: Ireland, Eng: England, Ger: Germany, Aus: Austria; Spa: Spain, Ita: Italy, UK: United Kingdom, Fra: France, Azo: Azores, Sao: São Tomé Island, Cam: Cameroon, Mal: Mali, Zam: Zambia, KLu: Luo from Kenia, KNa: Nandi from Kenia, Sen: Senegal, Gui: Guinea Bissau, Tar: Tarahumara, Gil: Native Americans from Gila River, Yup: Yu’pik from Alaska, Mit: Mixtec from Oaxaca, Zap: Zapotec from Oaxaca, Mix: Mixe from Oaxaca, Ser: Seri from Sonora, Nav: Navajo from New Mexico, HC: “Mexican Admixed controls” [26].

Principal component analysis (PCA) was performed to analyze the distribution of HLA-B alleles in human groups of the above-mentioned ancestral groups [26], Fig 1.

### Statistical analysis

Allele and genotype frequencies of HLA alleles, HLA blocks and haplotypes were compared using Chi-square (*X* ^2^) or Fisher exact test if appropriate; *p*-Values < 0.05 were considered statistically significant and they were corrected using Bonferroni (for allele frequencies, multiplying the original *p* value by the number of alleles displayed in the corresponding table) and Yates (for block and haplotype frequencies) tests; odds ratios with 95% confidence intervals (CI) were calculated to measure association strength (EPIINFO v7 software); numerical variables were analyzed using Student’s t-test. Hardy-Weinberg equilibrium (HWE) at each locus-by-locus level with 1 x 10^6^ steps in the Markov chain and 1 x 10^5^ dememorization steps. *p*-values ≤ 0.05 indicated a significant deviation from HWE.

## Results

### Demographic data and clinical characteristics

We included 159 SSc patients, 64 (40%) with dcSSc and 95 (60%) with lcSSc (Table 1). One hundred and forty seven (93%) were females and 12 were males (7%). There were more males in the dcSSc group (16%) than in the lcSSc group (2%, p = 0.004). Patients had a mean age ± SD of 45 ± 14.8 years at the inclusion to the study; 42 ± 13.9 in the dcSSc group and 47 ± 15.1 years in the lcSSc group (p = 0.04). Mean age at disease onset ± SD was 35 ± 14.2 years, without significant differences between the two disease subsets. Mean disease duration ± SD (from first symptom attributed to SSc) was 11 ± 9.7 years; 8 ± 6 years in the dcSSc group and 13 ± 1.1 years in the lcSSc group (p = 0.001).

**Table 1:** Demographic data and organ damage according to the modified Medsger’s Severity Scale.

*Variable*	*All SSc (n = 159)*	*dcSSc (n = 64) (40%)*	*lcSSc (n = 95) (60%)*	*p value*
Females (%)	147 (93)	54 (84)	93 (98)	0.004
Age (years±SD)	45 ± 14.8	42 ±13.9	47 ± 15.1	0.04
Age at onset (years±SD)	35 ± 14.1	34 ± 14.4	35 ± 13.9	0.59
Disease duration (years±SD)	11 ± 9.7	8.3 ± 6	13.1 ± 1.1	0.001
Organ involvement				
Peripheral vascular (%)	154 (97)	63 (98)	91 (96)	0.3
Joints (%)	115 (72)	51 (80)	64 (67)	0.08
Musculoskeletal (%)	35 (22)	17 (26)	18 (19)	0.2
Gastrointestinal tract (%)	101 (63)	47 (73)	54 (57)	0.03
ILD (%)	51 (32)	28 (44)	23 (24)	0.01
PAH (%)	24 (15)	12 (19)	12 (13)	0.3
Heart (%)	13 (8)	5 (8)	8 (8)	0.9
Kidney (%)	3 (2)	0	3 (3)	0.3

dcSSc: diffuse cutaneous systemic sclerosis, lcSSc: limited cutaneous systemic sclerosis, ILD: interstitial lung disease; PAH: pulmonary arterial hypertension.

Overall, 154 (97%) patients had peripheral vascular involvement; 32 (20%) had calcinosis; 115 (72%) had joint involvement; 35 (22%) had musculoskeletal damage (out of 107 patients that were evaluated for this variable); 101 (63%) had gastrointestinal involvement (102 patients evaluated); 51 (32%) had interstitial lung disease (115 patients evaluated); 24 (15%) patients had PAH (66 patients evaluated); 13 (8%) of patients had cardiac involvement (69 patients evaluated), and renal crisis was present in 3 (2%) patients.

### Autoantibodies

Table 2 shows SSc-associated autoantibodies in our patient population. Anticentromere antibodies were significantly more frequent in lcSSc patients (p = 0.005) and anti-Topoisomerase I antibodies were significantly more frequent in dcSSc patients (p = 0.0009). Other SSc-associated antibodies had lower frequencies and were equally distributed among dcSSc and lcSSc patients.

**Table 2:** Frequency of SSc-associated autoantibodies in our SSc patients.

*Autoantibody*	*All (n = 159)*	*dcSSc (n = 64)*	*lcSSc (n = 95)*	*P value*
	n (%)	n (%)	n (%)	
Anticentromere	47 (30)	11 (17)	36 (38)	0.005
Anti-Topoisomerase I	34 (21.4)	24 (38)	10 (10.5)	0.0009
Anti-U1-RNP	20 (13)	4 (6)	16 (17)	0.1
Anti-PM-Scl	12 (7)	5 (8)	7 (7.5)	0.9
Anti-RNA Pol III	1 (0.6)	0	1 (1)	0.4
Anti-Ku	12 (7)	4 (6)	8 (8)	0.6
Other	30 (19)	16 (25)	14 (15)	0.2
Negative ANA	3 (2)	0	3 (3)	0.3

dcSSc: diffuse cutaneous systemic sclerosis, lcSSc: limited cutaneous systemic sclerosis, Anti-RNA Pol III: anti-RNA polymerase I/III.

### HLA class I blocks and alleles associated to susceptibility or protection to SSc in Mexicans

The distribution of *HLA-A*, -*B* and -*C*, alleles, and HLA-C-B blocks frequencies in SSc patients and controls are listed in sections 1–4 of S1 Table. Significant deviations from the HWE were not detected for HLA class I loci.

We found three important associations of HLA class I genes with SSc that are detailed in Table 3:

**Table 3:** Relevant HLA allele and block associations in SSc patients compared to healthy controls.

HLA Block or allele	SSc (N = 316)	lcSSc (N = 188)	dcSSc (N = 128)	Controls (N = 468)	Comparissons, (pC value, Odds Ratio, 95% CI)	Association
	n (g.f.)	n (g.f.)	n (g.f.)	n (g.f.)		
C*07:*02*	*43 (0*.*1360)*	*33 (0*.*1750)*	*10 (0*.*0780)*	*97 (0*.*2070)*	*SSc vs Ctls (Yates pC = 0*.*01*, *OR = 0*.*60*, *95%CI = 0*.*40–0*.*89)*, *dcSSc vs Ctls (Yates pC = 0*.*001*, *OR = 0*.*32*, *95%CI = 0*.*16–0*.*64)*, *lcSSc vs Ctls (pC = ns)*, *dcSSc vs lcSSc (Yates pC = 0*.*02*, *OR = 0*.*39*, *95%CI = 0*.*18–0*.*84)*	*Protection to dcSSc*
B*39:*06*	*18 (0*.*0560)*	*15 (0*.*0790)*	*3 (0*.*0230)*	*32 (0*.*0680)*	*SSc vs Ctls (pC = ns)*, *dcSSc vs Ctls (pC = ns)*, *lcSSc vs Ctls (pC = ns)*, *dcSSC vs lcSSc (pC = ns)*	
B*39:*05*	*13 (0*.*0410)*	*10 (0*.*0530)*	*3 (0*.*0230)*	*37 (0*.*0790)*	*SSc vs Ctls (pC = ns)*, *dcSSc vs Ctls (Yates pC = 0*.*04*, *OR = 0*.*27*, *95%CI = 0*.*08–0*.*92)*, *lcSSc vs Ctls (pC = ns)*, *dcSSC vs lcSSc (pC = ns)*	
C*07:02-B*39:*06*	*18 (0*.*0569)*	*15 (0*.*0798)*	*3 (0*.*0234)*	*29 (0*.*0619)*	*SSc vs Ctls (pC = ns)*, *dcSSc vs Ctls (pC = ns)*, *lcSSc vs Ctls (pC = ns)*, *dcSSC vs lcSSc (pC = ns)*	
C*07:02-B*39:*05*	*12 (0*.*0379)*	*10 (0*.*0532)*	*2 (0*.*0156)*	*34 (0*.*0726)*	*SSc vs Ctls (pC = ns)*, *dcSSc vs Ctls (Yates pC = 0*.*02*, *OR = 0*.*20*, *95%CI = 0*.*04–0*.*85)*, *lcSSc vs Ctls (pC = ns)*, *dcSSC vs lcSSc (pC = ns)*	
C*07:02-B*39:06 + C*07:02-B*39:*05*	*30 (0*.*0949)*	*25 (0*.*1329)*	*5 (0*.*0390)*	*63 (0*.*1346)*	*SSc vs Ctls (pC = ns)*, *dcSSc vs Ctls (Yates pC = 0*.*004*, *OR = 0*.*26*, *95%CI = 0*.*10–0*.*66)*, *lcSSc vs Ctls (pC = ns)*, *dcSSc vs lcSSc (Yates pC = 0*.*009*, *OR = 0*.*26*, *95%CI = 0*.*09–0*.*71)*	*Protection to dcSSc*
***HLA Block or allele***						
C*07:*01*	*23 (0*.*0720)*	*11 (0*.*0580)*	*12 (0*.*0930)*	*25 (0*.*0530)*	*SSc vs Ctls (pC = ns)*, *dcSSc vs Ctls (pC = ns)*, *lcSSc vs Ctls (pC = ns)*, *dcSSc vs lcSSc (pC = ns)*	
B*08:*01*	*14 (0*.*0440)*	*8 (0*.*0420)*	*6 (0*.*0460)*	*3 (0*.*0060)*	*SSc vs Ctls (Yates pC = 0*.*0008*, *OR = 7*.*1*, *95%CI = 2*.*04–25*.*21)*, *dcSSc vs Ctls (Yates pC = 0*.*003*, *OR = 7*.*6*, *95%CI = 1*.*88–30*.*92)*, *lcSSc vs Ctls (Yates pC = 0*.*003*, *OR = 6*.*8; 95%CI = 1*.*8–26*.*25)*, *dcSSc vs lcSSc (pC = ns)*	*Susceptibility to SSc*
C*07:01-B*08:*01*	*11 (0*.*0348)*	*6 (0*.*0319)*	*5 (0*.*0391)*	*3 (0*.*0064)*	*SSc vs Ctls (Yates pC = 0*.*007*, *OR = 5*.*59*, *95%CI = 1*.*54–20*.*20)*, *dcSSc vs Ctls (Yates pC = 0*.*01*, *OR*, *6*.*3*, *95%CI = 1*.*48–26*.*73)*, *lcSSc vs Ctls (Yates pC = 0*.*03*, *OR = 5*.*1*, *95%CI = 1*.*26–20*.*65)*, *dcSSc vs lcSSc (pC = ns)*	*Susceptibility to SSc*
***HLA Block or allele***						
C*12:*03*	*18 (0*.*0560)*	*7 (0*.*0370)*	*9 (0*.*0700)*	*12 (0*.*0250)*	*SSc vs Ctls (Yates pC = 0*.*04*, *OR = 2*.*29*, *95% CI = 1*.*09–4*.*83)*, *dcSSc vs Ctls (Yates pC = 0*.*03*, *OR = 2*.*87*, *95%CI = 1*.*18–6*.*98)*, *lcSSc vs Ctls (pC = ns)*, *dcSSC vs lcSSc (pC = ns)*	
B*18:*01*	*12 (0*.*0370)*	*5 (0*.*0260)*	*7 (0*.*0540)*	*8 (0*.*0170)*	*SSc vs Ctls (pC = ns)*, *dcSSc vs Ctls (Yates pC = 0*.*03*, *OR = 3*.*32*, *95%CI = 1*.*18–9*.*35)*, *lcSSc vs Ctls (pC = ns)*, *dcSSC vs lcSSc (pC = ns)*	
C*12:03-B*18:*01*	*7 (0*.*0198)*	*1 (0*.*0053)*	*6 (0*.*0469)*	*2 (0*.*0042)*	*SSc vs Ctls (Yates pC = 0*.*04*, *OR = 5*.*27*, *95%CI = 1*.*08–25*.*57)*, *dcSSc vs Ctls (Yates pC = 0*.*001*, *OR = 11*.*46*, *95%CI = 2*.*28–57*.*48)*, *lcSSc vs Ctls (pC = ns)*, *dcSSC vs lcSSc (Yates pC = 0*.*03*, *OR = 9*.*1*, *95%CI = 1*.*09–77*.*32)*	*Susceptibility to dcSSc*
***HLA Block or allele***						
DRB1*11:*04*	*13 (0*.*0410)*	*3 (0*.*0150)*	*10 (0*.*0780)*	*8 (0*.*0170)*	*SSc vs Ctls (pC = ns)*, *dcSSc vs Ctls (Yates pC = 0*.*001*, *OR = 4*.*87*, *95%CI = 1*.*88–12*.*02)*, *lcSSc vs Ctls (pC = ns)*, *dcSSC vs lcSSc (Yates pC = 0*.*01*, *OR = 5*.*22*, *95%CI = 1*.*40–19*.*38)*	*Susceptibility to SSc*
DQB1*03:*01*	*46 (0*.*1450)*	*24 (0*.*1270)*	*22 (0*.*1710)*	*116 (0*.*2470)*	*SSc vs Ctls (Yates pC = 0*.*0007*, *OR = 0*.*51*, *95%CI = 0*.*35–0*.*75)*, *dcSSc vs Ctls (pC = ns)*, *lcSSc vs Ctls (Yates pC = 0*.*0009*, *OR = 0*.*44*, *95%CI = 0*.*27–0*.*71)*, *dcSSC vs lcSSc (pC = ns)*	*Protection to SSc (lcSSc)*
DRB1*11:04-DQB1*03:*01*	*11 (0*.*0348)*	*2 (0*.*0106)*	*9 (0*.*0703)*	*8 (0*.*0171)*	*SSc vs Ctls (pC = ns)*, *dcSSc vs Ctls (Yates pC = 0*.*001*, *OR = 4*.*87*, *95%CI = 1*.*64–11*.*51)*, *lcSSc vs Ctls (pC = ns)*, *dcSSC vs lcSSc (Yates pC = 0*.*01*, *OR = 7*.*03*, *95%CI = 1*.*49–33*.*10)*	*Susceptibility to SSc*
***HLA Block or allele***						
DRB1*01:*02*	*16 (0*.*0500)*	*11 (0*.*0580)*	*5 (0*.*0390)*	*11 (0*.*0230)*	*SSc vs Ctls (pC = ns)*, *dcSSc vs Ctls (pC = ns)*, *lcSSc vs Ctls (pC = 0*.*04*, *OR = 2*.*58*, *95%CI = 1*.*1–6*.*06)*, *dcSSc vs lcSSc (pC = ns)*	
DRB1*0101	*12 (0*.*0370)*	*10 (0*.*0530)*	*2 (0*.*0150)*	*9 (0*.*0190)*	*SSc vs Ctls (pC = ns)*, *dcSSc vs Ctls (pC = ns)*, *lcSSc vs Ctls (Yates pC = 0*.*03*, *OR = 2*.*8; 95%CI = 1*.*1–7*.*1)*, *dcSSC vs lcSSc (pC = ns)*	
DQB1*05:*01*	*32 ((0*.*1010)*	*21 (0*.*1110)*	*11 (0*.*0850)*	*32 (0*.*0680)*	*SSc vs Ctls (pC = ns)*, *dcSSc vs Ctls (pC = ns)*, *lcSSc vs Ctls (pC = ns)*, *dcSSc vs lcSSc (pC = ns)*	
DRB1*01:02-DQB1*05:01+DRB1*01:01-DQB1*0501	*28 (0*.*0800)*	*21 (0*.*1117)*	*7 (0*.*0546)*	*20 (0*.*0427)*	*SSc vs Ctls (Yates pC = 0*.*01*, *OR = 2*.*1*, *95%CI = 1*.*20–3*.*93)*, *dcSSc vs Ctls (pC = ns)*, *lcSSc vs Ctls (Yates pC = 0*.*001*, *OR = 2*.*80*, *95%CI = 1*.*48–5*.*32)*, *dcSSc vs lcSSc (pC = ns)*	*Susceptibiity to SSc*
***HLA Block or allele***						
DRB1*04:*04*	*17 (0*.*053)*	*15 (0*.*079)*	*2 (0*.*015)*	*31 (0*.*066)*	*SSc vs Ctls (pC = ns)*, *dcSSc vs Ctls (Yates pC = 0*.*04*, *OR = 0*.*2*, *95%CI = 0*.*05–0*.*94)*, *lcSSc vs Ctls (pC = ns)*, *dcSSC vs lcSSc (Yates pC = 0*.*02*, *OR = 0*.*18*, *95%CI = 0*.*04–0*.*81)*	
DQB1*03:*02*	*84 (0*.*26)*	*57 (0*.*303)*	*27 (0*.*21)*	*115 (0*.*245)*	*SSc vs Ctls (pC = ns)*, *dcSSc vs Ctls (pC = ns)*, *lcSSc vs Ctls (pC = ns)*, *dcSSC vs lcSSc (pC = ns)*	
DRB1*04:04/DQB1*03:*02*	*17 (0*.*053)*	*15 (0*.*079)*	*2 (0*.*015)*	*29 (0*.*062)*	*SSc vs Ctls (pC = ns)*, *dcSSc vs Ctls (pC = ns)*, *lcSSc vs Ctls (pC = ns)*, *dcSSC vs lcSSc (Yates pC = 0*.*02*, *OR = 0*.*1*, *95%CI = 0*.*01–0*.*8)*	
***HLA Block or allele***						
DRB1*14:*06*	*13 (0*.*0410)*	*8 (0*.*0420)*	*5 (0*.*0390)*	*47 (0*.*1000)*	*SSc vs Ctls (Yates pC = 0*.*002*, *OR = 0*.*30*, *95%CI = 0*.*20–0*.*72)*, *dcSSc vs Ctls (Yates pC = 0*.*04*, *OR = 0*.*36*, *95%CI = 0*.*14–0*.*93)*, *lcSSc vs Ctls (Yates pC = 0*.*02*, *OR = 0*.*39*, *95%CI = 0*.*18–0*.*85)*, *dcSSC vs lcSSc (pC = ns)*	*Protection to SSc*
DRB1*16:*02*	*7 (0*.*0220)*	*4 (0*.*0210)*	*3 (0*.*0230)*	*30 (0*.*0640)*	*SSc vs Ctls (Yates pC = 0*.*01*, *OR = 0*.*33*, *95%CI = 0*.*14–0*.*70)*, *dcSSc vs Ctls (pC = ns)*, *lcSSc vs Ctls (Yates pC = 0*.*04*, *OR = 0*.*31*, *95%CI = 0*.*11–0*.*91)*, *dcSSC vs lcSSc (pC = ns)*	
DRB1*14:*02*	*nd*	*nd*	*nd*	*11 (0*.*0235)*	*SSc vs Ctls (Yates pC = 0*.*03*, *OR = 0*.*12*, *95%CI = 0*.*01–0*.*93)*, *dcSSc vs Ctls (pC = 0*.*3)*, *lcSSc vs Ctls (pC = ns)*, *dcSSC vs lcSSc (pC = ns)*	
DQB1*03:*01*	*46 (0*.*1450)*	*24 (0*.*1270)*	*22 (0*.*1710)*	*116 (0*.*2470)*	*SSc vs Ctls (Yates pC = 0*.*0007*, *OR = 0*.*51*, *95%CI = 0*.*35–0*.*75)*, *dcSSc vs Ctls (pC = ns)*, *lcSSc vs Ctls (Yates pC = 0*.*0009*, *OR = 0*.*44*, *95%CI = 0*.*27–0*.*71)*, *dcSSC vs lcSSc (pC = ns)*	*Protection to SSc (lcSSc)*
DRB1*1406-DQB1*0301	*12 (0*.*0380)*	*8 (0*.*0426)*	*4 (0*.*0313)*	*46 (0*.*0983)*	*SSc vs Ctls (Yates pC = 0*.*002*, *OR = 0*.*36*, *95%CI = 0*.*18–0*.*69)*, *dcSSc vs Ctls (Yates pC = 0*.*02*, *OR = 0*.*29*, *95%CI = 0*.*10–0*.*83)*, *lcSSc vs Ctls (Yates pC = 0*.*02*, *OR = 0*.*40*, *95%CI = 0*.*18–0*.*88)*, *dcSSC vs lcSSc (pC = ns)*	*Protection to SSc*
DRB1*16:02-DQB1*0301	*7 (0*.*0222)*	*4 (0*.*0213)*	*3 (0*.*0234)*	*30 (0*.*0641)*	*SSc vs Ctls (Yates pC = 0*.*01*, *OR = 0*.*33*, *95%CI = 0*.*14–0*.*76)*, *dcSSc vs Ctls (pC = ns)*, *lcSSc vs Ctls (Yates pC = 0*.*04*, *OR = 0*.*3; 95%CI = 0*.*1–0*.*9)*, *dcSSC vs lcSSc (pC = ns)*	*Protection to SSc*
DRB1*14:02-DQB1*03:*01*	*nd*	*nd*	*nd*	*11 (0*.*0235)*	*SSc vs Ctls (Yates pC = 0*.*03*, *OR = 0*.*12*, *95%CI = 0*.*01–0*.*93)*, *dcSSc vs Ctls (pC = 0*.*3)*, *lcSSc vs Ctls (pC = ns)*, *dcSSC vs lcSSc (pC = ns)*	*Protection to SSc*
DRB1*1406-DQB1*0301 + DRB1*16:02-DQB1*0301 +DRB1*14:02-DQB1*03:*01*	*19 (0*.*0601)*	*12 (0*.*0638)*	*7 (0*.*0546)*	*87 (0*.*1858)*	*SSc vs Ctls (Yates pC = 0*.*000001*, *OR = 0*.*28*, *95%CI = 0*.*16–0*.*47)*, *dcSSc vs Ctls (Yates pC = 0*.*005*, *OR = 0*.*25*, *95%CI = 0*.*11–0*.*56)*, *lcSSc vs Ctls (Yates pC = 0*.*0001*, *OR = 0*.*29*, *95%CI = 0*.*15–0*.*56)*, *dcSSC vs lcSSc (pC = ns)*	*Protection to SSc*

**SSc:** Systemic sclerosis patients, **dcSSc:** diffuse cutaneous systemic sclerosis, **lcSSc:** limited cutaneous systemic sclerosis, **pC:** p corrected value, **OR:** odds ratio, **95% CI:** 95% confidence interval, **ns:** non-significant, **nd:** not detected.

A) We found a significant protective effect of the Amerindian allele HLA-C*07:02 for the susceptibility to dcSSc (pC = 0.001, OR 0.32 95%CI 0.1–0.6). This gene is part of the two Amerindian blocks HLA-C*07:02-B*39:06 and HLA-C*07:02-B*39:05, that have decreased frequency in the group of dcSSc patients when compared to lcSSc and healthy controls (Ctls) (sum of blocks: C*07:02-B*39:06 + C*07:02-B*39:05: dcSSc vs Ctls: pC = 0.004, OR = 0.26, 95%CI = 0.10–0.66, and dcSSc vs lcSSc pC = 0.009, OR = 0.26, 95%CI = 0.09–0.71). However, when the HLA-B*39:06 and B*39:05 alleles were analyzed separately, only HLA-B*39:05 exhibited marginal associations to dcSSc protection. These findings support that HLA-C*07:02 is mapping the protection gene for dcSSc.

B) We also found significant increase of the Caucasian HLA-C*07:01-B*08:01 block frequency (bf) in all SSc patients (bf 3.4%), as well as in both dcSSc (bf 3.9%) and lcSSc (bf 3.19%) groups, when compared to controls (bf 0.6%, SSc vs Ctls pC = 0.007, OR = 5.59, 95%CI = 1.54–20.2; dcSSc vs Ctls pC = 0.01, OR = 6.3, 95%CI = 1.48–26.73; lcSSc vs Ctls pC = 0.03, OR = 5.1, 95%CI = 1.26–20.65). The separate analysis of the alleles from this block showed increased frequency of HLA-B*08:01 in all SSc (gene frequency (gf) 4%), in dcSSc (gf = 4%) and in lcSSc (gf = 4.2%) patients when compared to controls (gf = 0.6%; SSc vs Ctls pC = 0.0008, OR = 7.1, 95%CI = 2.04–25.21; dcSSc vs Ctls pC = 0.003, OR = 7.6, 95%CI = 1.88–30.92; lcSSc vs Ctls pC = 0.003, OR = 6.8; 95%CI = 1.8–26.25). On the other hand, HLA-C*07:01 gene frequency was similar in all groups; this fact indicates that HLA-B*08:01 is the allele that explains the susceptibility association within this block.

C) Also, the Caucasian origin HLA-C*12:03-B*18:01 block was more common in SSc patients (1.9%) than in controls (0.4%, pC = 0.03, OR = 5.27, 95%CI = 1.08–25.27), particularly in dcSSc (4.7%, dcSSc vs Ctls pC = 0.001, OR = 11.46, 95%CI = 2.28–57) patients. The separated allele analysis revealed that both alleles HLA-C*12:03 (pC = 0.03, OR = 2.8, 95% CI = 1.18–6.98) and HLA-B*18:01 (pC = 0.03, OR = 3.2, 95%CI = 1.18–9.35) have an increased frequency in dcSSc patients compared to healthy controls, suggesting that the whole block is important as a susceptibility marker to dcSSc.

### HLA class II blocks and alleles associated to susceptibility or protection to SSc in Mexicans

The distribution of *HLA-DRB1 and –DQB1* alleles, and HLA-DRB1-DQB1 block frequencies in SSc patients and controls are listed in sections 5–7 of S1 Table. Significant deviations from HWE were not detected for HLA class II loci.

We found 4 important associations of HLA class II genes that are detailed in Table 3:

1. The Caucasian block DRB1*11:04-DQB1*03:01 was significantly increased in dcSSc (7%) but not in lcSSc patients (1%) when compared to controls (1.7%, dcSSc vs Ctls pC = 0.001, OR = 4.8, 95%CI = 1.64–11.51). The HLA-DRB1*11:04 allele was independently associated to dcSSc (gf = 7.8%, dcSSc vs Ctls pC = 0.001, OR = 4.87, 95%CI = 1.88–12.02), suggesting that this allele is mapping the susceptibility to dcSSc. Interestingly, the DQB1*03:01 allele, which is also part of this block with DRB1*11:04, was more frequent in healthy controls (see below) than in SSc patients, supporting a protective role of this allele in our population.
2. The combined frequency of the Caucasian blocks HLA-DRB1*01:02-DQB1*05:01 + DRB1*01:01-DQB1*05:01 was significantly associated to lcSSc (pC = 0.001, OR = 2.8, 95%CI = 1.48–5.32) when compared to healthy controls. This association depends on the presence of HLA-DRB1*01:01 (pC = 0.03, OR = 2.8; 95%CI = 1.1–7.1) and -DRB1*01:02 (pC = 0.04, OR = 2.58, 95%CI = 1.1–6.06), both alleles significantly more frequent in lcSSc patients than in controls. In contrast, the presence of HLA-DQB1*05:01 by itself was not associated to the disease (pC = ns).
3. The HLA-DRB1*04:04-DQB1*03:02 block was protective for dcSSc (bf = 1.5%, pC = 0.02, OR = 0.1, 95%CI = 0.04–0.81) when compared to lcSSc. The independent frequency of the DRB1*04:04 allele was significantly lower in dcSSc than in lcSSc patients (pC = 0.02, OR = 0.1, 95%CI = 0.04–0.81) and in controls (pC = 0.04, OR = 0.2, 95%CI = 0.05–0.94), suggesting that the protective effect is due to the DRB1*04:04 and not to the DQB1*03:02 allele that did not show significant association to any form of the disease.

### Protective effect of the DQB1*03:01 allele in SSc

We found three Amerindian blocks (HLA-DRB1*14:06-DQB1*03:01, DRB1*16:02-DQB1*03:01, and DRB1*14:02-DQB1*03:01) associated to protection for developing SSc (SSc vs controls: pC = 0.002, OR = 0.36, 95%CI = 0.18–0.69; pC = 0.01, OR = 0.33, 95%CI = 0.14–0.76; and pC = 0.03, OR = 0.12, 95%CI = 0.01–0.93, respectively). It is interesting that when we analyze every HLA-DRB1* allele from these blocks separately, they show a modest protective effect (SSc vs controls: DRB1*14:06: pC = 0.002, OR = 0.30, 95%CI = 0.20–0.72; DRB1*16:02: pC = 0.01, OR = 0.33, 95%CI = 0.14–0.70; and DRB1*14:02: pC = 0.03, OR = 0.12, 95%CI = 0.01–0.93), but when they are included in the block with DQB1*03:01, and when this allele is analyzed separately (SSc vs controls: pC = 0.0007, OR = 0.51, 95%CI = 0.35–0.75), the protective effect is enhanced, indicating that there is a protective effect that can be attributed to the DQB1*03:01 allele.

### Specific HLA class II alleles correlate with the presence of anti-topoisomerase autoantibodies

Antibody analysis revealed association of HLA-DRB1*08:02 (pC = 0.001, OR = 2.26, 95%CI = 1.2–2.4) and HLA-DQB1*04:02 alleles (pC = 0.000001, OR = 5.3, 95%CI = 2.8–10.1) to the presence of anti-Topoisomerase I antibody in SSc patients, while HLA-DQB1*03:02 was negatively associated to the presence of this autoantibody (pC = 0.006, OR = 0.34, 95%CI = 0.15–0.71). The presence of other autoantibodies did not exhibit any HLA association.

### HLA alleles and organ involvement in SSc patients

The presence of HLA-A*30:02 (p = 0.006, OR = 0.12, 95%CI = 0.01–0.68) was protective for joint-tendon involvement in all SSc patients. Severe and end-stage joint involvement, defined as finger to palm distance ≥ 4.0 cm, was associated to the presence of HLA-B*35:12 (p = 0.001, OR = 11.8, 95%CI = 2.7–47.2) and HLA-DQB1*06:03 (p = 0.005, OR = 12.9, 95%CI = 1.8–66) alleles. We found no HLA associations with other internal organ complications; however only a small percentage of patients in this study have had cardiac (8%) or kidney (2%) involvement (Data not shown).

### SSc patients and controls exhibit significant differences in the percentage of Amerindian, Caucasian and African genes

Mexican admixed individuals have a significant proportion of Amerindian and Caucasian genetic components, however, the admixture estimations revealed a lower proportion of Amerindian genetic component among SSc patients. (Section 8 of S1 Table). The admixture estimations using HLA-B revealed a contribution of 53.6% of Amerindian, 28.5% of Caucasian and 17.1% of African genes in SSc patients whereas in Controls we found 59.97% of Amerindian, 25.7% of Caucasian, and 14.5% of African genes contribution.

Interestingly, lcSSc patients had a distribution of 53.2% Amerindian, 33.5% Caucasian, and 13.2% African genes, while dcSSc patients had 53.3% Amerindian, 22.8% Caucasian and 23.7% of African genes. These findings show a significant increase in the percentage of Caucasian HLA-B genes and a reduced percentage of African HLA-B genes in patients with lcSSc.

Evidence of the distribution of immunogenetic diversity can be observed in the principal component analysis (PCA) plot (Fig 1), in which our SSc patients and healthy controls (HC) clusters together with Native American populations showing some subtle differences in the percentage of African an Caucasian genes among them.

## Discussion

Several case-control studies have shown that HLA class I and II alleles are associated to SSc [15–20]. Nevertheless, most studies did not take into account genetic admixture or HLA Class I and II blocks and haplotypes. In this report we determined how genetic admixture (Caucasian, African, Asian and Amerindian components) in Mexican SSc patients influenced the HLA associations to dcSSc and lcSSc subtypes. We found several examples of genetic associations of HLA class I and class II regions to SSc subtypes, antibodies and organ involvement.

Regarding HLA Class I, none of the associations described in this study: Amerindian origin allele HLA-C*07:02 (protective for dcSSc), Caucasian origin allele HLA-B*08:01 (associated to SSc susceptibility) and Caucasian origin block HLA-C*12:03-B*18:01 (associated to SSc susceptibility) had been described previously. Only the study by Gladman et al [16] had analyzed HLA Class I genes in Caucasian SSc patients.

Regarding HLA Class II associations:

1. Caucasian origin HLA-DRB1*11:04-DQB1*03:01 block was found associated to susceptibility to dcSSc. The susceptibility allele within this block is HLA-DRB1*11:04. This study confirms the findings by Arnett et al [15], Gladman et al [16] and Loubiere et al [36] that have found this association previously, while Simeon et al [37] and Reveille et al [38] found association of this allele (HLA-DRB1*11:04) to anti-topoisomerase antibodies in SSc patients; this association probably includes the same type of patients because this autoantibody is the most frequent in dcSSc patients [2].
2. Caucasian origin HLA-DRB1*01:01-DQB1*05:01 and HLA-DRB1*01:02-DQB1*05:01 blocks were associated to susceptibility to lcSSc; the analysis of these alleles maps the susceptibility to the DRB1*01 allele. Studies by Reveille et al [19], Simeon et al [37] and Gladman et al [16] have found HLA-DRB1*01 associated to disease susceptibility. Reveille et al [38], Kuwana et al [18] and Zhou et al [39] found association of HLA-DQB1*05:01 to the presence of anticentromere antibody, which is the most common autoantibody in limited cutaneous disease; these authors did not perform block or haplotype analysis so it is possible that in their patients, the HLA-DQB1*05:01 association is also due to linkage disequilibrium with a HLA-DRB1*01 allele.
3. The presence of HLA-DRB1*04:04-DQB1*03:02 block had a modest protective effect to the diffuse form of the disease. The protective effect could be mapped to the presence of DRB1*04:04 and is in concordance with the study by Louthrenoo et al in Thai population [40]; while Arnett et al [15] found HLA-DRB1*04:04 to be associated to the presence of anti-RNA polymerase III autoantibody in a mixed population from USA with a possibly high Caucasian component. This finding is interesting because this is a very uncommon antibody in Mexican admixed SSc patients (2% of Mexican SSc patients) [13], hence, the rarity of this allele (DRB1*04:04) and this antibody (anti-RNA polymerase III) in our dcSSc population support the theory of the influence of the immunogenetic background in the different clinical expression of the disease in populations from different ethnic groups.
4. Amerindian origin blocks containing HLA-DQB1*03:01 showed a protective effect to both forms of the disease. This finding contrasts with previous studies by Arnett et al [15], Tikly et al [41] and by Reveille et al [38] in different ethnic groups that showed this allele to be associated to SSc susceptibility, or to the presence of anti-topoisomerase I antibody in Japanese patients [42].

Many studies have reported associations of HLA alleles with SSc-associated autoantibodies [14–20,38–41,41,42]. In this report, we found only one Amerindian HLA class II DNA block (DRB1*08:02-DQB1*04:02) associated to the presence of anti-Topoisomerase I antibody (Section 7 of S1 Table). Since the statistical significance for this association was higher for DQB1*04:02 than for DRB1*08:02, then we can conclude that the former is the most relevant allele within this block.

In Table 4 we show the most significant HLA class II associations with specific autoantibody profiles in different ethnicities. Special attention should be focused on the study by Arnett et al 2010 [15] that also found DQB1*04:02 and DRB1*08:02 to be associated to the presence of anti-Topoisomerase I antibody in Hispanics from Texas. These findings in both studies involving patients with similar but not identical ethnic background support the idea of the influence of immunogenetic factors on the clinical and autoantibody phenotype. Also, the presence of HLA-DRB1*11:04 has been associated in Caucasians and Hispanics [15, 37, 43] to anti-Topoisomerase I antibody. In the present study this allele, was more prevalent in patients with dcSSc (the phenotype more associated to anti-Topoisomerase I antibody) than in those with lcSSc.

**Table 4:** HLA class II genes associations with specific autoantibodies in SSc.

HLA class II allele/haplotype	Autoantibody Association	Population	Reference
DRB1*15:02	Anticentromere	Chinese	He D, et al. [45]
DRB1*16:02	Anti-Topoisomerase I	Chinese	He D, et al. [45]
DPB1*04	Anticentromere	Chinese	Wang J, et al. [46]
DQB1*05:01	Anticentromere	Chinese	Zhou XD, et al. [39]
DQB1*06:01	Anti-Topoisomerase I	Chinese	Zhou XD, et al. [39]
DRB1*15:02	Anti-Topoisomerase I	Thai	Louthrenoo W, et al. [40]
DRB1*01	Anticentromere	Caucasian French	Azzouz DF, et al. [47]
DRB1*11	Anti-Topoisomerase I	Caucasian French	Azzouz DF, et al. [47]
DRB1*11:04	**Anti-Topoisomerase I**	Caucasian Spanish, Italian	Beretta L, et al. [43]
DRB1*08:04	Anti-U3-RNP	African American	Sharif R, et al. [48]
DRB1*13:02-DQB1*06:04 haplotype	Anti-U3-RNP	African American	Arnett FC, et al. [49]
DRB1*1104, DQB1*03:01, DPB1*1301,DQB1 alleles with a glycine at position 26	**Anti-Topoisomerase I**	Caucasian	Arnett FC, et al. [15]
DRB1*08:04, DRB1*11:04, DQB1*03:01	**Anti-Topoisomerase I**	African-American	Arnett FC, et al. [15]
DRB1*08:02, DRB1*11:04, DQA1*04:01/*03:01, DQB1*04:02	**Anti-Topoisomerase I**	Hispanics	Arnett FC, et al. [15]
DRB1*04:04, DRB1*11:04, DQB1*03:01	anti-RNAP I/III	Caucasian NA	Arnett FC, et al. [15]
DRB1*08:04, DQA1*05:01, DQB1*03:01	anti-RNAP I/III	African American	Arnett FC, et al. [15]
DRB1*11:04, DQA1*05:01, and DQB1*03:01	anti-RNAP I/III	Hispanics	Arnett FC, et al. [15]
DRB1*01:01, *0401, *04:04, *08:01, DQA1*01:01, DQB1*03:01, *03:02, *05:01, DQB1 26	Anticentromere	Caucasian NA	Arnett FC, et al. [15]
DRB1*04:07	Anticentromere	Hispanics	Arnett FC, et al. [15]
DRB1*04:05, DRB4*01, and DQB1*04:01	anti-RNAP I/III	Japanese	Kuwana M, et al. [50]
DRB3*02	anti-RNAP I/III	Caucasian	Kuwana M, et al. [50]
DRB1*15:02; DQB1*06:01 haplotype	Anti-Topoisomerase I	Japanese	Takeuchi, F, et al. [51]
DPB1*13:01	Anti-Topoisomerase I	Caucasian UK	Gilchrist FC, et al. [52]
DRB1*04, DRB1*08, DQB1 alleles with a glycine at position 26	Anticentromere	Caucasian UK	Gilchrist FC, et al. [52]
DRB1*15:02-DQB1*06:01-DPB1*09:01 haplotype	Anti-Topoisomerase I	Japanese	Kuwana M, et al. [17]
DRB1*04:01/*08:02-DQB1*03:02 haplotype	anti-U1-RNP	Japanese	Kuwana M, et al. [17]
DRB1*01:01-DQB1*05:01-DPB1*04:02	Anticentromere	Japanese	Kuwana M, et al. [17]
DRB1*11:01	Anti-Topoisomerase I	Caucasian NA	Kuwana M, et al. [53]
DRB1*11:04	**Anti-Topoisomerase I**	African American	Kuwana M, et al. [53]
DRB1*15:02	Anti-Topoisomerase I	Japanese	Kuwana M, et al. [53]
DRB1*16:02	Anti-Topoisomerase I	Native American	Kuwana M, et al. [53]
**DR11**	Anti-Th/To, **anti-Topoisomerase I**	Caucasian NA	Falkner D, et al. [54]
DQB1*05:01	Anticentromere	Japanese	Kuwana M, et al. [18]
DQB1-05:01	Anticentromere	Northern European, Eastern European, Mediterranean, and non-Caucasian	Morel PA, et al. [55]
DR11	Anti-Topoisomerase I	Caucasian NA	Morel PA, et al. [56]
DRB1*11:04	**Anti-Topoisomerase I**	Caucasian Spanish	Simeón CP, et al. [37]
DRB1*01 and DQB1*05	Anticentromere	Caucasian Spanish	Simeón CP, et al. [37]
DPB1*13:01 and DRB1*15	Anti-Topoisomerase I	Black South Africans	Tikly M, et al. [41]
DRB1*11:01 and DQB1*06:03/14	Anti-U3-RNP	Black South Africans	Tikly M, et al. [41]
HLA-DR5(DRB1*11:01-*11:04), DRB3*02:02, DQw3	Anti-Topoisomerase I	Caucasians	Revielle JD, et al. [19]
HLA-DQB1*03:01	Anti-Topoisomerase I	American Blacks	Revielle JD, et al. [19]
DQB1 alleles Leu-26 negative, HLA-DR5 (DRw11), Dw13(DRB1*04:03, *04:07)	Anticentromere	Caucasian	Revielle JD, et al. [20]
DQB1*04:02 (DRB1*08:02-DQB1*04:02 Haplotype)	**Anti-Topoisomerase I**	Mexican Admixed	Rodriguez-Reyna TS, et al. Present study

anti-RNAP I/III: anti-RNA polymerase I/III.

Regarding the analysis of internal organ complications, we report here the association of HLA-B*35:12 and HLA-DQB1*06:03 alleles to severe joint involvement; only the study by Tikly et al [41] had found an association of HLA-DQB1*06:03 to a SSc feature (the presence of anti-fibrillarin antibodies). However, these associations should be considered as preliminary, since they involve a small number of cases for each analysis.

The inconsistency in HLA association studies performed in SSc patients with different ethnic backgrounds, suggests that the variation in the frequency of HLA alleles and HLA CEHs between major or ancestral populations from different geographical areas may explain in part the differential susceptibility to develop autoimmune diseases and the differences in their clinical presentations amongst ethnic groups; this is possibly a result of genetic fixity of HLA alleles due to past selective processes or infectious and parasitic diseases developed in different environments. Thus it is possible that the differences of HLA-SSc associations observed between our and previous studies in patients with Mexican and Central-American ancestry [15] are the result of subtle differences in the genetic diversity of HLA genes, like those that have been previously reported between Mexicans from the north, central and south areas from Mexico [44].

Our study has some limitations, including its relatively small sample size, which was restricted by the rigorous criteria for case and control definition regarding clinical and ethnic background characteristics. With these strictly defined cases, we were able to confirm some of the previous associations of HLA class II genes to SSc, and we identified novel associations of these genes with susceptibility to SSc and SSc-specific autoantibodies, with a statistical power greater than 80%. Another limitation was the lack of a validation cohort. It is important to highlight that our SSc cohort was established in 2006, using international standards, and yearly homogeneous follow-up is performed by specialists in the field. Our Institute is a referral center for SSc patients in Mexico; furthermore, to our knowledge, there is not another comparable SSc cohort in Mexico that can be used to validate the genetic data. Also, we must consider that this study was performed in a prevalent cohort; hence, selection bias may have influenced the results by excluding patients with very severe disease that could have died within the first years of the disease and were not included in the analysis.

In summary, this is the first report that uses high resolution typing of HLA class I and II alleles and haplotype diversity in a well-characterized cohort of Mexican patients with SSc. The HLA HLA-C*12:03-B*18:01 and HLA-C*07:01-B*08:01 haplotypes are associated to susceptibility to dcSSc whereas C*07:02-B*39:05, C*07:02-B*39:06 and DQB1*03:01 are associated to protection to SSc. The allele HLA-DQB1*04:02, was associated to anti-Topoisomerase I antibody and we found alleles associated to internal organ damage. The admixture estimations revealed a lower proportion of Amerindian genetic component among SSc patients, suggesting that Amerindian genes do not represent an additional risk for developing SSc. These findings support the contribution of HLA class I and class II genes and their haplotypes in the protection and susceptibility to SSc, in its different clinical presentations as well as in SSc-specific autoantibodies in Mexican admixed individuals.

## Supporting Information

S1 Table Frequencies of HLA-A, HLA-B and HLA-C alleles, HLA-C-B blocks, HLA-DRB1 and HLA-DQB1 alleles, HLA-DRB1-DQB1 blocks and HLA conserved extended haplotypes in Mexican SSc patients and healthy controls. (DOCX)
