# Supplementary material for: HLA Class I and II Blocks Are Associated to Susceptibility, Clinical Subtypes and Autoantibodies in Mexican Systemic Sclerosis (SSc) Patients
Source: PLoS One. 2015 May 20;10(5):e0126727. doi: 10.1371/journal.pone.0126727 (PMC4439150; doi:10.1371/journal.pone.0126727)
Supplement: S1 Table — (DOCX) [file pone.0126727.s001.docx]

| **Section 1. Frequencies of HLA-A alleles in Mexican SSc patients and healthy controls.** | | | | | | | | | | | |
| --- | --- | --- | --- | --- | --- | --- | --- | --- | --- | --- | --- |
|  | **Total SSc (N=316)** | |  | **Limited SSc (N=188)** | |  | **Diffuse SSc (N=128)** | |  | **Controls (N=468)** | |
| **Allele** | **n** | **g.f.** |  | **n** | **g.f.** |  | **n** | **g.f.** |  | **n** | **g.f.** |
| A*02:01 | 68 | 0.2150 |  | 47 | 0.2500 |  | 21 | 0.1640 |  | 107 | 0.2280 |
| A*24:02 | 46 | 0.1450 |  | 26 | 0.1380 |  | 20 | 0.1560 |  | 79 | 0.1680 |
| A*68:01 | 23 | 0.0720 |  | 10 | 0.0530 |  | 13 | 0.1010 |  | 37 | 0.0790 |
| A*31:01 | 22 | 0.0690 |  | 11 | 0.0580 |  | 11 | 0.0850 |  | 37 | 0.0790 |
| A*02:06 | 19 | 0.0600 |  | 9 | 0.0470 |  | 10 | 0.0780 |  | 45 | 0.0960 |
| A*01:01 | 18 | 0.0560 |  | 9 | 0.0470 |  | 9 | 0.0700 |  | 17 | 0.0360 |
| A*68:02 | 13 | 0.0410 |  | 11 | 0.0580 |  | 2 | 0.0150 |  | 14 | 0.0290 |
| A*11:01 | 11 | 0.0340 |  | 8 | 0.0420 |  | 3 | 0.0230 |  | 10 | 0.0210 |
| A*26:01 | 9 | 0.0280 |  | 3 | 0.0150 |  | 6 | 0.0460 |  | 9 | 0.0190 |
| A*30:02 | 8 | 0.0250 |  | 6 | 0.0310 |  | 2 | 0.0150 |  | 7 | 0.0150 |
| A*29:02 | 8 | 0.0250 |  | 7 | 0.0370 |  | 1 | 0.0070 |  | 12 | 0.0250 |
| A*03:01 | 8 | 0.0250 |  | 5 | 0.0260 |  | 3 | 0.0230 |  | 15 | 0.0320 |
| A*68:03 | 8 | 0.0250 |  | 5 | 0.0260 |  | 3 | 0.0230 |  | 16 | 0.0340 |
| A*33:01 | 7 | 0.0220 |  | 4 | 0.0210 |  | 3 | 0.0230 |  | 6 | 0.0120 |
| A*32:01 | 6 | 0.0180 |  | 4 | 0.0210 |  | 2 | 0.0150 |  | 4 | 0.0080 |
| A*23:01 | 6 | 0.0180 |  | 4 | 0.0210 |  | 2 | 0.0150 |  | 8 | 0.0170 |
| A*30:01 | 2 | 0.0060 |  | 2 | 0.0100 |  | ND |  |  | 6 | 0.0120 |
| A*74:01 | 2 | 0.0060 |  | 2 | 0.0100 |  | ND |  |  | 1 | 0.0020 |
| A*68:05 | 2 | 0.0060 |  | ND |  |  | 2 | 0.0150 |  | 3 | 0.0060 |
| A*25:01 | 2 | 0.0060 |  | 1 | 0.0050 |  | 1 | 0.0070 |  | 3 | 0.0060 |
| A*69:01 | 2 | 0.0060 |  | 1 | 0.0050 |  | 1 | 0.0070 |  | ND |  |
| A*02:02 | 1 | 0.0030 |  | ND |  |  | 1 | 0.0070 |  | 1 | 0.0020 |
| A*02:05 | 1 | 0.0030 |  | 1 | 0.0050 |  | ND |  |  | 8 | 0.0170 |
| A*02:44 | 1 | 0.0030 |  | ND |  |  | 1 | 0.0070 |  | ND |  |
| A*24:22 | 1 | 0.0030 |  | ND |  |  | 1 | 0.0070 |  | ND |  |
| A*24:25 | 1 | 0.0030 |  | 1 | 0.0050 |  | ND |  |  | 3 | 0.0060 |
| A*29:01 | 1 | 0.0030 |  | ND |  |  | 1 | 0.0070 |  | ND |  |
| A*30:04 | 1 | 0.0030 |  | 1 | 0.0050 |  | ND |  |  | 1 | 0.0020 |
| A*30:10 | 1 | 0.0030 |  | 1 | 0.0050 |  | ND |  |  | ND |  |
| A*34:01 | 1 | 0.0030 |  | 1 | 0.0050 |  | ND |  |  | 1 | 0.0020 |
| A*34:02 | 1 | 0.0030 |  | ND |  |  | 1 | 0.0070 |  | 1 | 0.0020 |
| A*01:02 | ND |  |  | ND |  |  | ND |  |  | 2 | 0.0040 |
| A*24:03 | ND |  |  | ND |  |  | ND |  |  | ND |  |

| **Section 2. Frequencies of HLA-B alleles in Mexican SSc patients and healthy controls.** | | | | | | | | | | | |
| --- | --- | --- | --- | --- | --- | --- | --- | --- | --- | --- | --- |
|  | **Total SSC (N=316)** | |  | **Limited SSc (N=188)** | |  | **Diffuse SSc (N=128)** | |  | **Controls (N=468)** | |
| **Allele** | **n** | **g.f.** |  | **n** | **g.f.** |  | **n** | **g.f.** |  | **n** | **g.f.** |
| B*35:01 | 21 | 0.0660 |  | 11 | 0.0580 |  | 10 | 0.0780 |  | 27 | 0.0570 |
| B*35:12 | 20 | 0.0630 |  | 12 | 0.0630 |  | 8 | 0.0620 |  | 18 | 0.0380 |
| B*40:02 | 20 | 0.0630 |  | 9 | 0.0470 |  | 11 | 0.0850 |  | 25 | 0.0530 |
| B*51:01 | 18 | 0.0560 |  | 10 | 0.0530 |  | 8 | 0.0620 |  | 28 | 0.0590 |
| B*39:06 | 18 | 0.0560 |  | 15 | 0.0790 |  | 3 | 0.0230 |  | 32 | 0.0680 |
| B*35:17 | 17 | 0.0530 |  | 12 | 0.0630 |  | 5 | 0.0390 |  | 18 | 0.0380 |
| B*08:01 | 14 | 0.0440 |  | 8 | 0.0420 |  | 6 | 0.0460 |  | 3 | 0.0060 |
| B*39:05 | 13 | 0.0410 |  | 10 | 0.0530 |  | 3 | 0.0230 |  | 37 | 0.0790 |
| B*14:02 | 13 | 0.0410 |  | 9 | 0.0470 |  | 4 | 0.0310 |  | 15 | 0.0320 |
| B*18:01 | 12 | 0.0370 |  | 5 | 0.0260 |  | 7 | 0.0540 |  | 8 | 0.0170 |
| B*15:15 | 12 | 0.0370 |  | 8 | 0.0420 |  | 4 | 0.0310 |  | 15 | 0.0320 |
| B*52:01 | 10 | 0.0310 |  | 5 | 0.0260 |  | 5 | 0.0390 |  | 10 | 0.0210 |
| B*45:01 | 8 | 0.0250 |  | 4 | 0.0210 |  | 4 | 0.0310 |  | 3 | 0.0060 |
| B*38:01 | 8 | 0.0250 |  | 3 | 0.0150 |  | 5 | 0.0390 |  | 6 | 0.0120 |
| B*44:03 | 7 | 0.0220 |  | 5 | 0.0260 |  | 2 | 0.0150 |  | 13 | 0.0270 |
| B*44:02 | 7 | 0.0220 |  | 7 | 0.0370 |  | ND |  |  | 5 | 0.0100 |
| B*49:01 | 6 | 0.0180 |  | 2 | 0.0100 |  | 4 | 0.0310 |  | 9 | 0.0190 |
| B*15:01 | 6 | 0.0180 |  | 5 | 0.0260 |  | 1 | 0.0070 |  | 10 | 0.0210 |
| B*48:01 | 6 | 0.0180 |  | 2 | 0.0100 |  | 4 | 0.0310 |  | 20 | 0.0420 |
| B*53:01 | 6 | 0.0180 |  | 4 | 0.0210 |  | 2 | 0.0150 |  | 6 | 0.0120 |
| B*07:02 | 5 | 0.0150 |  | 4 | 0.0210 |  | 1 | 0.0070 |  | 19 | 0.0400 |
| B*15:30 | 5 | 0.0150 |  | 3 | 0.0150 |  | 2 | 0.0150 |  | 8 | 0.0170 |
| B*27:05 | 5 | 0.0150 |  | 4 | 0.0210 |  | 1 | 0.0070 |  | 3 | 0.0060 |
| B*39:01 | 5 | 0.0150 |  | 5 | 0.0260 |  | ND |  |  | 5 | 0.0100 |
| B*50:01 | 3 | 0.0090 |  | 2 | 0.0100 |  | 1 | 0.0070 |  | 4 | 0.0080 |
| B*14:01 | 3 | 0.0090 |  | 2 | 0.0100 |  | 1 | 0.0070 |  | 4 | 0.0080 |
| B*40:05 | 3 | 0.0090 |  | 2 | 0.0100 |  | 1 | 0.0070 |  | 5 | 0.0100 |
| B*40:11 | 3 | 0.0090 |  | 2 | 0.0100 |  | 1 | 0.0070 |  | ND |  |
| B*41:01 | 3 | 0.0090 |  | 1 | 0.0050 |  | 2 | 0.0150 |  | 5 | 0.0100 |
| B*51:02 | 3 | 0.0090 |  | ND |  |  | 3 | 0.0230 |  | 2 | 0.0040 |
| B*35:14 | 3 | 0.0090 |  | 2 | 0.0100 |  | 1 | 0.0070 |  | 7 | 0.0150 |
| B*35:02 | 2 | 0.0060 |  | ND |  |  | 2 | 0.0150 |  | 2 | 0.0040 |
| B*15:10 | 2 | 0.0060 |  | 1 | 0.0050 |  | 1 | 0.0070 |  | 1 | 0.0020 |
| B*15:17 | 2 | 0.0060 |  | 2 | 0.0100 |  | ND |  |  | 3 | 0.0060 |
| B*35:08 | 2 | 0.0060 |  | 2 | 0.0100 |  | ND |  |  | 3 | 0.0060 |
| B*35:43 | 2 | 0.0060 |  | 1 | 0.0050 |  | 1 | 0.0070 |  | 9 | 0.0190 |
| B*39:08 | 2 | 0.0060 |  | 1 | 0.0050 |  | 1 | 0.0070 |  | 3 | 0.0060 |
| B*40:01 | 2 | 0.0060 |  | 2 | 0.0100 |  | ND |  |  | ND |  |
| B*55:01 | 2 | 0.0060 |  | ND |  |  | 2 | 0.0150 |  | 3 | 0.0060 |
| B*56:01 | 2 | 0.0060 |  | ND |  |  | 2 | 0.0150 |  | 1 | 0.0020 |
| B*58:01 | 2 | 0.0060 |  | 1 | 0.0050 |  | 1 | 0.0070 |  | 3 | 0.0060 |
| B*42:01 | 2 | 0.0060 |  | 1 | 0.0050 |  | 1 | 0.0070 |  | ND |  |
| B*39:02 | 1 | 0.0030 |  | ND |  |  | 1 | 0.0070 |  | ND |  |
| B*13:02 | 1 | 0.0030 |  | ND |  |  | 1 | 0.0070 |  | 6 | 0.0120 |
| B*35:03 | 1 | 0.0030 |  | 1 | 0.0050 |  | ND |  |  | 5 | 0.0100 |
| B*50:02 | 1 | 0.0030 |  | 1 | 0.0050 |  | ND |  |  | ND |  |
| B*07:05 | 1 | 0.0030 |  | 1 | 0.0050 |  | ND |  |  | ND |  |
| B*15:16 | 1 | 0.0030 |  | ND |  |  | 1 | 0.0070 |  | 1 | 0.0020 |
| B*15:48 | 1 | 0.0030 |  | ND |  |  | 1 | 0.0070 |  | ND |  |
| B*27:03 | 1 | 0.0030 |  | 1 | 0.0050 |  | ND |  |  | 1 | 0.0020 |
| B*35:16 | 1 | 0.0030 |  | ND |  |  | ND |  |  | 3 | 0.0060 |
| B*40:06 | 1 | 0.0030 |  | 1 | 0.0050 |  | ND |  |  | ND |  |
| B*44:05 | 1 | 0.0030 |  | ND |  |  | 1 | 0.0070 |  | ND |  |
| B*51:14 | 1 | 0.0030 |  | 1 | 0.0050 |  | ND | 0.0070 |  | ND |  |
| B*58:02 | 1 | 0.0030 |  | ND |  |  | 1 | 0.0070 |  | 6 | 0.0120 |
| B*35:02 | 1 | 0.0030 |  | 1 | 0.0050 |  | ND |  |  | 2 | 0.0040 |
| B*57:01 | ND |  |  | ND |  |  | ND |  |  | 7 | 0.0150 |

| **Section 3. Frequencies of HLA-C alleles in Mexican SSc patients and healthy controls.** | | | | | | | | | | | |
| --- | --- | --- | --- | --- | --- | --- | --- | --- | --- | --- | --- |
|  | **Total SSc (N=316)** | |  | **Limited SSc (N=188)** | |  | **Diffuse SSc (N=128)** | |  | **Controls (N=468)** | |
| **Allele** | **n** | **g.f.** |  | **n** | **g.f.** |  | **n** | **g.f.** |  | **n** | **g.f.** |
| C*04:01 | 71 | 0.2240 |  | 43 | 0.2280 |  | 28 | 0.2180 |  | 87 | 0.1850 |
| C*07:02 | 43 | 0.1360 |  | 33 | 0.1750 |  | 10 | 0.0780 |  | 97 | 0.2070 |
| C*01:02 | 29 | 0.0910 |  | 20 | 0.1060 |  | 9 | 0.0700 |  | 42 | 0.0890 |
| C*03:04 | 25 | 0.0790 |  | 14 | 0.0740 |  | 11 | 0.0850 |  | 31 | 0.0660 |
| C*07:01 | 23 | 0.0720 |  | 11 | 0.0580 |  | 12 | 0.0930 |  | 25 | 0.0530 |
| C*12:03 | 18 | 0.0560 |  | 7 | 0.0370 |  | 9 | 0.0700 |  | 12 | 0.0250 |
| C*08:02 | 15 | 0.0470 |  | 10 | 0.0530 |  | 5 | 0.0390 |  | 19 | 0.0400 |
| C*08:01 | 13 | 0.0410 |  | 5 | 0.0260 |  | 8 | 0.0620 |  | 22 | 0.0470 |
| C*06:02 | 11 | 0.0340 |  | 4 | 0.0210 |  | 7 | 0.0540 |  | 28 | 0.0590 |
| C*05:01 | 11 | 0.0340 |  | 10 | 0.0530 |  | 1 | 0.0070 |  | 10 | 0.0210 |
| C*15:02 | 10 | 0.0310 |  | 7 | 0.0370 |  | 3 | 0.0230 |  | 9 | 0.0190 |
| C*16:01 | 8 | 0.0250 |  | 5 | 0.0260 |  | 3 | 0.0230 |  | 12 | 0.0250 |
| C*03:06 | 7 | 0.0220 |  | 1 | 0.0050 |  | 6 | 0.0460 |  | ND |  |
| C*02:02 | 7 | 0.0220 |  | 4 | 0.0210 |  | 3 | 0.0230 |  | 6 | 0.0120 |
| C*03:03 | 6 | 0.0180 |  | 3 | 0.0150 |  | 3 | 0.0230 |  | 14 | 0.0290 |
| C*14:02 | 4 | 0.0120 |  | 2 | 0.0100 |  | 2 | 0.0150 |  | 4 | 0.0080 |
| C*12:02 | 3 | 0.0090 |  | 2 | 0.0100 |  | 1 | 0.0070 |  | 2 | 0.0040 |
| C*03:05 | 3 | 0.0090 |  | 1 | 0.0050 |  | 2 | 0.0150 |  | 16 | 0.0340 |
| C*15:09 | 3 | 0.0090 |  | 2 | 0.0100 |  | 1 | 0.0070 |  | 11 | 0.0230 |
| C*17:01 | 3 | 0.0090 |  | 1 | 0.0050 |  | 2 | 0.0150 |  | 1 | 0.0020 |
| C*05:09 | 1 | 0.0030 |  | 1 | 0.0050 |  | ND |  |  | ND |  |
| C*07:04 | 1 | 0.0030 |  | ND |  |  | 1 | 0.0070 |  | 1 | 0.0020 |
| C*08:03 | 1 | 0.0030 |  | 1 | 0.0050 |  | ND |  |  | 4 | 0.0080 |
| C*14:03 | 1 | 0.0030 |  | ND |  |  | 1 | 0.0070 |  | ND |  |
| C*15:05 | 1 | 0.0030 |  | 1 | 0.0050 |  | ND |  |  | 1 | 0.0020 |

| **Section 4. Frequencies of HLA-C-B blocks in Mexican SSc patients and healthy controls.** | | | | | | | | | | | | | | | | | | |
| --- | --- | --- | --- | --- | --- | --- | --- | --- | --- | --- | --- | --- | --- | --- | --- | --- | --- | --- |
|  |  |  |  | **SSc (N=316)** | |  |  | **Limited (N=188)** | | |  | **Diffuse (N=128)** | |  |  | **Controls (N=468)** | | |
|  | **HLA-C/-B block** | |  | **n** | **H.F.** | **D'** |  | **n** | **H.F.** | **D'** |  | **n** | **H.F.** | **D'** |  | **n** | **H.F.** | **D'** |
| **Amerindian** | C*04:01 | B*35:12 |  | 20 | 0.0632 | 1.0000 |  | 12 | 0.0638 | 1.0000 |  | 8 | 0.0625 | 1.0000 |  | 16 | 0.0341 | 0.8632 |
|  | C*07:02 | B*39:06 |  | 18 | 0.0569 | 1.0000 |  | 15 | 0.0798 | 1.0000 |  | 3 | 0.0234 | 1.0000 |  | 29 | 0.0619 | 0.8025 |
|  | C*04:01 | B*35:17 |  | 17 | 0.0537 | 1.0000 |  | 12 | 0.0638 | 1.0000 |  | 5 | 0.0391 | 1.0000 |  | 17 | 0.0363 | 1.0000 |
|  | C*07:02 | B*39:05 |  | 12 | 0.0379 | 0.9110 |  | 10 | 0.0532 | 1.0000 |  | 2 | 0.0156 | 0.6384 |  | 34 | 0.0726 | 0.8975 |
|  | C*15:02 | B*40:02 |  | 12 | 0.0319 | 0.4662 |  | 4 | 0.0213 | 0.5499 |  | 1 | 0.0078 | 0.2707 |  | ND |  |  |
|  | C*01:02 | B*15:15 |  | 9 | 0.0289 | 1.0000 |  | 8 | 0.0426 | 1.0000 |  | 4 | 0.0313 | 1.0000 |  | 13 | 0.0277 | 0.8534 |
|  | C*03:06 | B*40:02 |  | 6 | 0.0189 | 0.8475 |  | 1 | 0.0053 | 1.0000 |  | 5 | 0.0391 | 0.8177 |  | ND |  |  |
|  | C*03:04 | B*40:02 |  | 5 | 0.0158 | 0.1856 |  | 3 | 0.0160 | 0.2797 |  | 2 | 0.0156 | 0.1049 |  | 11 | 0.0235 | 0.4196 |
|  | C*01:02 | B*15:30 |  | 5 | 0.0158 | 1.0000 |  | 3 | 0.0160 | 1.0000 |  | 2 | 0.0156 | 1.0000 |  | 8 | 0.0170 | 1.0000 |
|  | C*01:02 | B*15:01 |  | 5 | 0.0158 | 0.8165 |  | 5 | 0.0266 | 1.0000 |  | ND |  |  |  | 7 | 0.0149 | 0.6701 |
|  | C*08:01 | B*48:01 |  | 4 | 0.0126 | 0.6524 |  | 1 | 0.0053 | 0.4863 |  | 3 | 0.0234 | 0.7333 |  | 15 | 0.0320 | 0.7376 |
|  | C*03:05 | B*40:02 |  | 3 | 0.0094 | 1.0000 |  | 1 | 0.0053 | 1.0000 |  | 2 | 0.0156 | 1.0000 |  | 10 | 0.0213 | 0.6045 |
|  | C*02:02 | B*27:05 |  | 3 | 0.0094 | 0.5909 |  | 2 | 0.0106 | 0.4891 |  | 1 | 0.0078 | 1.0000 |  | 2 | 0.0042 | 0.6623 |
|  | C*08:01 | B*51:02 |  | 3 | 0.0094 | 1.0000 |  | ND |  |  |  | 3 | 0.0234 | 1.0000 |  | 2 | 0.0042 | 1.0000 |
|  | C*03:04 | B*35:01 |  | 2 | 0.0063 | 0.0175 |  | 2 | 0.0106 | 0.1160 |  | ND |  |  |  | 1 | 0.0021 | -0.4456 |
|  | C*03:04 | B*40:05 |  | 2 | 0.0063 | 0.6380 |  | 2 | 0.0106 | 1.0000 |  | ND |  |  |  | ND |  |  |
|  | C*04:01 | B*35:03 |  | 1 | 0.0031 | 1.0000 |  | 1 | 0.0053 | 1.0000 |  | ND |  |  |  | 4 | 0.0085 | 0.7538 |
|  | C*07:01 | B*15:17 |  | 1 | 0.0031 | 0.4608 |  | 1 | 0.0053 | 0.4689 |  | ND |  |  |  | 3 | 0.0064 | 1.0000 |
|  | C*08:03 | B*48:01 |  | 1 | 0.0031 | 1.0000 |  | 1 | 0.0053 | 1.0000 |  | ND |  |  |  | 3 | 0.0064 | 0.7387 |
|  | C*07:02 | B*35:01 |  | 1 | 0.0031 | -0.6501 |  | ND |  |  |  | 1 | 0.0078 | 0.0237 |  | 1 | 0.0021 | -0.8228 |
|  | C*03:04 | B*51:01 |  | 1 | 0.0031 | -0.2978 |  | ND |  |  |  | 1 | 0.0078 | 0.0427 |  | 1 | 0.0021 | -0.4654 |
|  | Other |  |  | 0 |  |  |  | 0 |  |  |  | 0 |  |  |  | 17 | 0.0085 |  |
|  |  | **Total** |  | **131** | **0.4077** |  |  | **84** | **0.4467** |  |  | **43** | **0.3358** |  |  | **194** | **0.4133** |  |
| **Caucasian** | C*07:01 | B*08:01 |  | 11 | 0.0348 | 0.7689 |  | 6 | 0.0319 | 0.7345 |  | 5 | 0.0391 | 0.8161 |  | 3 | 0.0064 | 1.0000 |
|  | C*12:03 | B*18:01 |  | 7 | 0.0198 | 0.6170 |  | 1 | 0.0053 | 0.2210 |  | 6 | 0.0469 | 0.8463 |  | 2 | 0.0042 | 0.2301 |
|  | C*12:03 | B*38:01 |  | 6 | 0.0189 | 0.7367 |  | 3 | 0.0160 | 1.0000 |  | 3 | 0.0234 | 0.5698 |  | 6 | 0.0128 | 1.0000 |
|  | C*05:01 | B*44:02 |  | 6 | 0.0189 | 0.8520 |  | 6 | 0.0319 | 0.8491 |  | ND |  |  |  | 4 | 0.0085 | 0.7956 |
|  | C*07:02 | B*07:02 |  | 4 | 0.0126 | 0.7685 |  | 3 | 0.0160 | 0.6968 |  | 1 | 0.0078 | 1.0000 |  | 15 | 0.0320 | 0.7893 |
|  | C*05:01 | B*18:01 |  | 3 | 0.0094 | 0.2465 |  | 3 | 0.0160 | 0.7360 |  | ND |  |  |  | 5 | 0.0106 | 0.6167 |
|  | C*06:02 | B*50:01 |  | 3 | 0.0094 | 1.0000 |  | 2 | 0.0106 | 1.0000 |  | 1 | 0.0078 | 1.0000 |  | 4 | 0.0085 | 1.0000 |
|  | C*16:01 | B*44:03 |  | 2 | 0.0053 | 0.3330 |  | 1 | 0.0053 | 0.1781 |  | 1 | 0.0078 | 0.4880 |  | 8 | 0.0170 | 0.6571 |
|  | C*07:01 | B*18:01 |  | 2 | 0.0063 | 0.0196 |  | ND |  |  |  | 1 | 0.0078 | 0.0542 |  | ND |  |  |
|  | C*04:01 | B*44:03 |  | 1 | 0.0031 | 0.1256 |  | 1 | 0.0053 | 0.1256 |  | ND |  |  |  | 3 | 0.0064 | 0.0533 |
|  | C*17:01 | B*41:01 |  | 1 | 0.0031 | 0.3269 |  | ND |  |  |  | 1 | 0.0078 | 0.4921 |  | 1 | 0.0021 | 1.0000 |
|  | C*03:04 | B*15:01 |  | 1 | 0.0031 | 0.0951 |  | ND |  |  |  | 1 | 0.0078 | 1.0000 |  | 2 | 0.0042 | 0.1427 |
|  | Other |  |  | 0 |  |  |  | 0 |  |  |  | 0 |  |  |  | 11 | 0.0722 |  |
|  |  | **Total** |  | **47** | **0.1447** |  |  | **26** | **0.1383** |  |  | **20** | **0.1562** |  |  | **64** | **0.1359** |  |
| **Caucasian** | C*04:01 | B*35:01 |  | 17 | 0.0539 | 0.7543 |  | 9 | 0.0479 | 0.7643 |  | 8 | 0.0625 | 0.7440 |  | 15 | 0.0320 | 0.4530 |
| **Shared** | C*08:02 | B*14:02 |  | 12 | 0.0379 | 1.0000 |  | 8 | 0.0426 | 1.0000 |  | 4 | 0.0313 | 1.0000 |  | 11 | 0.0235 | 0.7219 |
|  | C*07:01 | B*49:01 |  | 6 | 0.0189 | 1.0000 |  | 2 | 0.0106 | 1.0000 |  | 4 | 0.0313 | 1.0000 |  | 6 | 0.0128 | 0.6477 |
|  | C*03:03 | B*52:01 |  | 6 | 0.0189 | 1.0000 |  | 3 | 0.0160 | 1.0000 |  | 3 | 0.0234 | 1.0000 |  | 6 | 0.0128 | 0.5876 |
|  | C*15:02 | B*51:01 |  | 4 | 0.0126 | 0.3638 |  | 2 | 0.0106 | 0.2456 |  | 2 | 0.0156 | 0.6444 |  | 9 | 0.0192 | 1.0000 |
|  | C*08:02 | B*14:01 |  | 3 | 0.0094 | 1.0000 |  | 2 | 0.0106 | 1.0000 |  | 1 | 0.0078 | 1.0000 |  | 4 | 0.0085 | 1.0000 |
|  | C*12:02 | B*52:01 |  | 3 | 0.0094 | 1.0000 |  | 2 | 0.0106 | 1.0000 |  | 1 | 0.0078 | 1.0000 |  | 2 | 0.0042 | 1.0000 |
|  | C*01:02 | B*35:43 |  | 2 | 0.0063 | 1.0000 |  | 1 | 0.0053 | 1.0000 |  | 1 | 0.0078 | 1.0000 |  | 9 | 0.0192 | 1.0000 |
|  | C*07:01 | B*41:01 |  | 2 | 0.0063 | 0.6405 |  | 1 | 0.0053 | 1.0000 |  | 1 | 0.0078 | 0.4483 |  | 4 | 0.0085 | 0.7886 |
|  | C*15:09 | B*51:01 |  | 2 | 0.0063 | 0.7359 |  | 1 | 0.0053 | 0.4719 |  | 1 | 0.0078 | 1.0000 |  | 9 | 0.0192 | 0.8065 |
|  | C*02:02 | B*40:02 |  | 1 | 0.0031 | 0.2707 |  | ND |  |  |  | 1 | 0.0078 | 0.2707 |  | 1 | 0.0021 | 0.1212 |
|  | C*06:02 | B*13:02 |  | 1 | 0.0031 | 1.0000 |  | ND |  |  |  | 1 | 0.0078 | 1.0000 |  | 5 | 0.0106 | 0.8226 |
|  | Other |  |  | 0 |  |  |  | 0 |  |  |  | 0 |  |  |  | 0 | 0 |  |
|  |  | **Total** |  | **59** | **0.1861** |  |  | **31** | **0.1648** |  |  | **28** | **0.2187** |  |  | **81** | **0.1406** |  |
| **African** | C*04:01 | B*53:01 |  | 6 | 0.0189 | 1.0000 |  | 4 | 0.0213 | 1.0000 |  | 2 | 0.0156 | 1.0000 |  | 6 | 0.0128 | 1.0000 |
|  | C*06:02 | B*45:01 |  | 4 | 0.0126 | 0.4820 |  | 1 | 0.0053 | 0.2337 |  | 3 | 0.0234 | 0.7355 |  | 2 | 0.0042 | 0.6453 |
|  | C*16:01 | B*45:01 |  | 4 | 0.0126 | 0.4870 |  | 3 | 0.0160 | 0.7432 |  | 1 | 0.0078 | 0.3118 |  | 1 | 0.0021 | 0.3156 |
|  | C*06:02 | B*58:02 |  | 1 | 0.0031 | 1.0000 |  | ND |  |  |  | 1 | 0.0078 | 1.0000 |  | 6 | 0.0128 | 1.0000 |
|  | C*02:02 | B*27:03 |  | 1 | 0.0031 | 1.0000 |  | 1 | 0.0053 | 1.0000 |  | ND |  |  |  | 1 | 0.0021 | 1.0000 |
|  | C*14:02 | B*15:16 |  | 1 | 0.0031 | 1.0000 |  | ND |  |  |  | 1 | 0.0078 | 1.0000 |  | 1 | 0.0021 | 1.0000 |
|  | Other |  |  |  |  |  |  |  |  |  |  |  |  |  |  | 9 | 0.0190 |  |
|  |  | **Total** |  | **17** | **0.0534** |  |  | **9** | **0.0479** |  |  | **8** | **0.0624** |  |  | **26** | **0.0551** |  |
| **Asian** | C*14:02 | B*51:01 |  | 3 | 0.0094 | 0.7349 |  | 2 | 0.0106 | 1.0000 |  | 1 | 0.0078 | 0.4667 |  | 3 | 0.0064 | 0.7339 |
|  | C*01:02 | B*55:01 |  | 1 | 0.0031 | 0.4495 |  | ND |  |  |  | 1 | 0.0078 | 0.4622 |  | 1 | 0.0021 | 0.2670 |
|  | Other |  |  | 0 |  |  |  | 0 |  |  |  | 0 |  |  |  | 12 | 0.0253 |  |
|  |  | **Total** |  | **4** | **0.0125** |  |  | **2** | **0.0106** |  |  | **2** | **0.0156** |  |  | **16** | **0.0338** |  |
| **Unknown** | C*08:01 | B*51:01 |  | 6 | 0.0189 | 0.4290 |  | 4 | 0.0213 | 0.7888 |  | 2 | 0.0156 | 0.2000 |  | 1 | 0.0021 | -0.2468 |
|  | C*04:01 | B*35:02 |  | 3 | 0.0094 | 1.0000 |  | 1 | 0.0053 | 1.0000 |  | 2 | 0.0156 | 1.0000 |  | ND |  |  |
|  | C*03:04 | B*40:11 |  | 3 | 0.0094 | 1.0000 |  | 2 | 0.0106 | 1.0000 |  | 1 | 0.0078 | 1.0000 |  | ND |  |  |
|  | C*04:01 | B*35:14 |  | 2 | 0.0063 | 0.5701 |  | 1 | 0.0053 | 0.3517 |  | 1 | 0.0078 | 1.0000 |  | 6 | 0.0128 | 1.0000 |
|  | C*07:01 | B*58:01 |  | 2 | 0.0063 | 1.0000 |  | 1 | 0.0053 | 1.0000 |  | 1 | 0.0078 | 1.0000 |  | 1 | 0.0021 | 0.2954 |
|  | C*03:04 | B*15:10 |  | 2 | 0.0063 | 1.0000 |  | 1 | 0.0053 | 1.0000 |  | 1 | 0.0078 | 1.0000 |  | ND |  |  |
|  | C*01:02 | B*27:05 |  | 2 | 0.0063 | 0.3394 |  | 2 | 0.0106 | 0.4405 |  | ND |  |  |  | ND |  |  |
|  | C*03:04 | B*40:01 |  | 2 | 0.0063 | 1.0000 |  | 2 | 0.0106 | 1.0000 |  | ND |  |  |  | ND |  |  |
|  | C*17:01 | B*42:01 |  | 2 | 0.0063 | 1.0000 |  | 1 | 0.0053 | 1.0000 |  | 1 | 0.0078 | 1.0000 |  | ND |  |  |
|  | C*12:03 | B*44:03 |  | 2 | 0.0063 | 0.3768 |  | 2 | 0.0106 | 0.3768 |  | ND |  |  |  | ND |  |  |
|  | C*12:03 | B*39:01 |  | 1 | 0.0031 | 0.1573 |  | 1 | 0.0053 | 0.2210 |  | ND |  |  |  | ND |  |  |
|  | C*15:09 | B*51:14 |  | 1 | 0.0031 | 1.0000 |  | 1 | 0.0053 | 1.0000 |  | ND |  |  |  | ND |  |  |
|  | C*07:02 | B*39:01 |  | 1 | 0.0031 | 0.0740 |  | 1 | 0.0053 | 0.0903 |  | ND |  |  |  | ND |  |  |
|  | C*04:01 | B*35:08 |  | 1 | 0.0031 | 0.3517 |  | 1 | 0.0053 | 0.3517 |  | ND |  |  |  | 3 | 0.0064 | 1.0000 |
|  | C*07:02 | B*51:01 |  | 1 | 0.0031 | -0.5917 |  | ND |  |  |  | 1 | 0.0078 | 0.0509 |  | 2 | 0.0042 | -0.6583 |
|  | C*15:05 | B*07:02 |  | 1 | 0.0031 | 1.0000 |  | 1 | 0.0053 | 1.0000 |  | ND |  |  |  | 1 | 0.0021 | 1.0000 |
|  | C*03:04 | B*39:01 |  | 1 | 0.0031 | 0.1313 |  | 1 | 0.0053 | 0.1897 |  | ND |  |  |  | 1 | 0.0021 | 0.1427 |
|  | C*16:01 | B*35:01 |  | 1 | 0.0031 | 0.0627 |  | ND |  |  |  | 1 | 0.0078 | 0.2768 |  | 1 | 0.0021 | 0.0267 |
|  | C*07:02 | B*44:03 |  | 1 | 0.0031 | 0.0079 |  | 1 | 0.0053 | 0.0297 |  | ND |  |  |  | ND |  |  |
|  | C*04:01 | B*39:01 |  | 1 | 0.0031 | -0.1099 |  | ND |  |  |  | 1 | 0.0078 | 1.0000 |  | ND |  |  |
|  | C*07:02 | B*08:01 |  | 1 | 0.0031 | -0.4751 |  | 1 | 0.0053 | -0.2879 |  | ND |  |  |  | ND |  |  |
|  | C*03:04 | B*08:01 |  | 1 | 0.0031 | -0.0971 |  | ND |  |  |  | 1 | 0.0078 | 0.0883 |  | ND |  |  |
|  | C*05:01 | B*15:17 |  | 1 | 0.0031 | 0.4820 |  | 1 | 0.0053 | 0.4719 |  | ND |  |  |  | ND |  |  |
|  | C*04:01 | B*51:01 |  | 1 | 0.0031 | -0.7527 |  | 1 | 0.0053 | -0.5628 |  | ND |  |  |  | ND |  |  |
|  | C*01:02 | B*08:01 |  | 1 | 0.0031 | -0.2217 |  | 1 | 0.0053 | 0.0208 |  | ND |  |  |  | ND |  |  |
|  | C*03:04 | B*35:14 |  | 1 | 0.0031 | 0.2761 |  | 1 | 0.0053 | 0.4598 |  | ND |  |  |  | ND |  |  |
|  | C*15:02 | B*40:06 |  | 1 | 0.0031 | 1.0000 |  | 1 | 0.0053 | 1.0000 |  | ND |  |  |  | ND |  |  |
|  | C*07:02 | B*39:08 |  | 1 | 0.0031 | 1.0000 |  | 1 | 0.0053 | 1.0000 |  | 1 | 0.0078 | 1.0000 |  | ND |  |  |
|  | C*03:06 | B*56:01 |  | 1 | 0.0031 | 0.4887 |  | ND |  |  |  | 1 | 0.0078 | 0.4754 |  | ND |  |  |
|  | C*07:02 | B*07:05 |  | 1 | 0.0031 | 1.0000 |  | 1 | 0.0053 | 1.0000 |  | ND |  |  |  | ND |  |  |
|  | C*07:04 | B*56:01 |  | 1 | 0.0031 | 1.0000 |  | ND |  |  |  | 1 | 0.0078 | 1.0000 |  | ND |  |  |
|  | C*03:04 | B*52:01 |  | 1 | 0.0031 | 0.0227 |  | ND |  |  |  | 1 | 0.0078 | 0.1248 |  | ND |  |  |
|  | C*03:04 | B*39:02 |  | 1 | 0.0031 | 1.0000 |  | ND |  |  |  | 1 | 0.0078 | 1.0000 |  | ND |  |  |
|  | C*02:02 | B*44:05 |  | 1 | 0.0031 | 1.0000 |  | ND |  |  |  | 1 | 0.0078 | 1.0000 |  | ND |  |  |
|  | C*03:04 | B*55:01 |  | 1 | 0.0031 | 0.4570 |  | ND |  |  |  | 1 | 0.0078 | 0.4530 |  | ND |  |  |
|  | C*01:02 | B*15:48 |  | 1 | 0.0031 | 1.0000 |  | ND |  |  |  | 1 | 0.0078 | 1.0000 |  | ND |  |  |
|  | C*14:03 | B*44:03 |  | 1 | 0.0031 | 1.0000 |  | ND |  |  |  | 1 | 0.0078 | 1.0000 |  | ND |  |  |
|  | C*16:01 | B*39:01 |  | 1 | 0.0031 | 0.1792 |  | 1 | 0.0053 | 0.2295 |  | ND |  |  |  | ND |  |  |
|  | C*06:02 | B*50:02 |  | 1 | 0.0031 | 1.0000 |  | 1 | 0.0053 | 1.0000 |  | ND |  |  |  | ND |  |  |
|  | C*05:09 | B*44:02 |  | 1 | 0.0031 | 1.0000 |  | 1 | 0.0053 | 1.0000 |  | ND |  |  |  | ND |  |  |
|  | C*02:02 | B*35:08 |  | 1 | 0.0031 | 0.4891 |  | 1 | 0.0053 | 0.4891 |  | ND |  |  |  | ND |  |  |
|  | C*05:01 | B*38:01 |  | 1 | 0.0031 | 0.0934 |  | ND |  |  |  | 1 | 0.0078 | 1.0000 |  | ND |  |  |
|  | C*06:02 | B*38:01 |  | 1 | 0.0031 | 0.0934 |  | ND |  |  |  | 1 | 0.0078 | 0.1537 |  | ND |  |  |
|  | C*03:04 | B*39:05 |  | 1 | 0.0031 | -0.0277 | 77 | ND |  |  |  | 1 | 0.0078 | 0.2707 |  | ND |  |  |
|  | C*07:02 | B*40:05 |  | 1 | 0.0031 | 0.2283 |  | ND |  |  |  | 1 | 0.0078 | 1.0000 |  | 1 | 0.0021 | -0.0433 |
|  | C*04:01 | B*48:01 |  | 1 | 0.0031 | -0.2582 |  | ND |  |  |  | 1 | 0.0078 | 0.0400 |  | ND |  |  |
|  | Other |  |  | 0 |  |  |  | 0 |  |  |  | 0 |  |  |  | 70 | 0.2004 |  |
|  |  | **Total** |  | **62** | **0.1934** |  |  | **36** | **0.1909** |  |  | **27** | **0.2106** |  |  | **87** | **0.2194** |  |

| **Section 5. Frequencies of HLA-DRB1 alleles in Mexican SSc patients and healthy controls.** | | | | | | | | | | | |
| --- | --- | --- | --- | --- | --- | --- | --- | --- | --- | --- | --- |
|  | **Total SSc (N=316)** | |  | **Limited SSc (N=188)** | |  | **Diffuse SSc (N=128)** | |  | **Controls (N=468)** | |
| **Allele** | **n** | **g.f.** |  | **n** | **g.f.** |  | **n** | **g.f.** |  | **n** | **g.f.** |
| DRB1*08:02 | 69 | 0.2180 |  | 35 | 0.1860 |  | 34 | 0.2650 |  | 91 | 0.1940 |
| DRB1*04:07 | 44 | 0.1390 |  | 27 | 0.1430 |  | 17 | 0.1320 |  | 55 | 0.1170 |
| DRB1*04:04 | 17 | 0.0530 |  | 15 | 0.0790 |  | 2 | 0.0150 |  | 31 | 0.0660 |
| DRB1*01:02 | 16 | 0.0500 |  | 11 | 0.0580 |  | 5 | 0.0390 |  | 11 | 0.0230 |
| DRB1*03:01 | 14 | 0.0440 |  | 9 | 0.0470 |  | 5 | 0.0390 |  | 15 | 0.0320 |
| DRB1*14:06 | 13 | 0.0410 |  | 8 | 0.0420 |  | 5 | 0.0390 |  | 47 | 0.1000 |
| DRB1*11:04 | 13 | 0.0410 |  | 3 | 0.0150 |  | 10 | 0.0780 |  | 8 | 0.0170 |
| DRB1*07:01 | 12 | 0.0370 |  | 7 | 0.0370 |  | 5 | 0.0390 |  | 33 | 0.0700 |
| DRB1*01:01 | 12 | 0.0370 |  | 10 | 0.0530 |  | 2 | 0.0150 |  | 9 | 0.0190 |
| DRB1*04:03 | 11 | 0.0340 |  | 7 | 0.0370 |  | 4 | 0.0310 |  | 10 | 0.0210 |
| DRB1*15:01 | 10 | 0.0310 |  | 6 | 0.0310 |  | 4 | 0.0310 |  | 17 | 0.0360 |
| DRB1*04:11 | 9 | 0.0280 |  | 5 | 0.0260 |  | 4 | 0.0310 |  | 9 | 0.0190 |
| DRB1*04:05 | 7 | 0.0220 |  | 5 | 0.0260 |  | 2 | 0.0150 |  | 1 | 0.0020 |
| DRB1*13:01 | 7 | 0.0220 |  | 4 | 0.0210 |  | 3 | 0.0230 |  | 12 | 0.0250 |
| DRB1*16:02 | 7 | 0.0220 |  | 4 | 0.0210 |  | 3 | 0.0230 |  | 30 | 0.0640 |
| DRB1*11:01 | 5 | 0.0150 |  | 4 | 0.0210 |  | 1 | 0.0070 |  | 6 | 0.0120 |
| DRB1*13:02 | 6 | 0.0180 |  | 3 | 0.0150 |  | 3 | 0.0230 |  | 10 | 0.0210 |
| DRB1*08:01 | 5 | 0.0150 |  | 3 | 0.0150 |  | 2 | 0.0150 |  | 1 | 0.0020 |
| DRB1*15:02 | 5 | 0.0150 |  | 4 | 0.0210 |  | 1 | 0.0070 |  | 5 | 0.0100 |
| DRB1*04:01 | 4 | 0.0120 |  | 3 | 0.0150 |  | 1 | 0.0070 |  | 3 | 0.0060 |
| DRB1*14:01 | 4 | 0.0120 |  | 3 | 0.0150 |  | 1 | 0.0070 |  | 8 | 0.0170 |
| DRB1*11:02 | 4 | 0.0120 |  | 1 | 0.0050 |  | 3 | 0.0230 |  | 4 | 0.0080 |
| DRB1*15:03 | 4 | 0.0120 |  | 2 | 0.0100 |  | 2 | 0.0150 |  | 1 | 0.0020 |
| DRB1*13:04 | 3 | 0.0090 |  | 2 | 0.0100 |  | 1 | 0.0070 |  | 1 | 0.0020 |
| DRB1*08:04 | 3 | 0.0090 |  | 1 | 0.0050 |  | 2 | 0.0150 |  | 2 | 0.0040 |
| DRB1*10:01 | 2 | 0.0060 |  | ND |  |  | 2 | 0.0150 |  | 6 | 0.0120 |
| DRB1*04:02 | 2 | 0.0060 |  | 1 | 0.0050 |  | 1 | 0.0070 |  | 10 | 0.0210 |
| DRB1*04:06 | 2 | 0.0060 |  | 2 | 0.0100 |  | ND |  |  | ND |  |
| DRB1*11:03 | 1 | 0.0030 |  | 1 | 0.0050 |  | ND |  |  | ND |  |
| DRB1*01:03 | 1 | 0.0030 |  | ND |  |  | 1 | 0.0070 |  | 3 | 0.0060 |
| DRB1*03:02 | 1 | 0.0030 |  | ND |  |  | 1 | 0.0070 |  | 1 | 0.0020 |
| DRB1*03:15 | 1 | 0.0030 |  | 1 | 0.0050 |  | ND |  |  | ND |  |
| DRB1*04:08 | 1 | 0.0030 |  | ND |  |  | 1 | 0.0070 |  | 1 | 0.0020 |
| DRB1*12:01 | 1 | 0.0030 |  | 1 | 0.0050 |  | ND |  |  | 2 | 0.0040 |
| DRB1*16:01 | ND |  |  | ND |  |  | ND |  |  | 2 | 0.0040 |

| **Section 6. Frequencies of HLA-DQB1 alleles in Mexican SSc patients and healthy controls.** | | | | | | | | | | | |
| --- | --- | --- | --- | --- | --- | --- | --- | --- | --- | --- | --- |
|  | **Total SSc (N=316)** | |  | **Limited SSc (N=188)** | |  | **Diffuse SSc (N=128)** | |  | **Controls (N=468)** | |
| **Allele** | **n** | **g.f.** |  | **n** | **g.f.** |  | **n** | **g.f.** |  | **n** | **g.f.** |
| DQB1*03:02 | 84 | 0.2650 |  | 57 | 0.3030 |  | 27 | 0.2100 |  | 115 | 0.2450 |
| DQB1*04:02 | 81 | 0.2560 |  | 42 | 0.2230 |  | 39 | 0.3040 |  | 96 | 0.2050 |
| DQB1*03:01 | 46 | 0.1450 |  | 24 | 0.1270 |  | 22 | 0.1710 |  | 116 | 0.2470 |
| DQB1*05:01 | 32 | 0.1010 |  | 21 | 0.1110 |  | 11 | 0.0850 |  | 32 | 0.0680 |
| DQB1*02:01 | 15 | 0.0470 |  | 10 | 0.0530 |  | 5 | 0.0390 |  | 15 | 0.0320 |
| DQB1*06:02 | 12 | 0.0370 |  | 7 | 0.0370 |  | 5 | 0.0390 |  | 17 | 0.0360 |
| DQB1*02:02 | 11 | 0.0340 |  | 7 | 0.0370 |  | 4 | 0.0310 |  | 28 | 0.0590 |
| DQB1*06:03 | 10 | 0.0310 |  | 4 | 0.0210 |  | 6 | 0.0460 |  | 7 | 0.0150 |
| DQB1*03:19 | 6 | 0.0180 |  | 3 | 0.0150 |  | 3 | 0.0230 |  | 5 | 0.0100 |
| DQB1*06:01 | 5 | 0.0150 |  | 4 | 0.0210 |  | 1 | 0.0070 |  | 5 | 0.0100 |
| DQB1*05:03 | 4 | 0.0120 |  | 3 | 0.0150 |  | 1 | 0.0070 |  | 8 | 0.0170 |
| DQB1*06:09 | 3 | 0.0090 |  | 2 | 0.0100 |  | 1 | 0.0070 |  | ND |  |
| DQB1*05:02 | 2 | 0.0060 |  | 2 | 0.0100 |  | ND |  |  | 3 | 0.0060 |
| DQB1*06:04 | 2 | 0.0060 |  | 1 | 0.0050 |  | 1 | 0.0070 |  | 10 | 0.0210 |
| DQB1*03:03 | 2 | 0.0060 |  | 1 | 0.0050 |  | 1 | 0.0070 |  | 10 | 0.0210 |
| DQB1*03:05 | 1 | 0.0030 |  | ND |  |  | 1 | 0.0070 |  | ND |  |

| **Section 7. Frequencies of HLA-DRB1-DQB1 blocks in Mexican SSc patients and healthy controls.** | | | | | | | | | | | | | | | | | | |
| --- | --- | --- | --- | --- | --- | --- | --- | --- | --- | --- | --- | --- | --- | --- | --- | --- | --- | --- |
|  |  |  |  | **SSc (N=316)** | |  |  | **Limited (N=188)** | | |  | **Diffuse (N=128)** | | |  | **Controls (N=468)** | | |
|  | HLA-DRB1/-DQB1 block | |  | n | H.F. | D' |  | n | H.F. | D' |  | n | H.F. | D' |  | n | H.F. | D' |
| **Amerindian** | DRB1*08:02 | DQB1*04:02 |  | 68 | 0.2152 | 0.9805 |  | 35 | 0.1862 | 1.0000 |  | 33 | 0.2578 | 0.9577 |  | 89 | 0.1902 | 0.9723 |
|  | DRB1*04:07 | DQB1*03:02 |  | 43 | 0.1361 | 0.9690 |  | 27 | 0.1436 | 1.0000 |  | 16 | 0.1250 | 0.9255 |  | 54 | 0.1153 | 0.9518 |
|  | DRB1*14:06 | DQB1*03:01 |  | 12 | 0.0380 | 0.9100 |  | 8 | 0.0426 | 1.0000 |  | 4 | 0.0313 | 0.7585 |  | 46 | 0.0983 | 0.9717 |
|  | DRB1*16:02 | DQB1*03:01 |  | 7 | 0.0222 | 1.0000 |  | 4 | 0.0213 | 1.0000 |  | 3 | 0.0234 | 1.0000 |  | 30 | 0.0641 | 1.0000 |
|  | DRB1*04:11 | DQB1*03:02 |  | 5 | 0.0158 | 0.3946 |  | 3 | 0.0160 | 0.4260 |  | 2 | 0.0156 | 0.3663 |  | 8 | 0.0171 | 0.8526 |
|  | DRB1*04:11 | DQB1*04:02 |  | 4 | 0.0127 | 0.2530 |  | 2 | 0.0106 | 0.2274 |  | 2 | 0.0156 | 0.2809 |  | 1 | 0.0022 | -0.4595 |
|  | DRB1*04:07 | DQB1*04:02 |  | 1 | 0.0032 | -0.9113 |  | ND |  |  |  | 1 | 0.0078 | -0.8069 |  | ND |  |  |
|  | Other |  |  | 0 |  |  |  | 0 |  |  |  | 0 |  |  |  | 12 | 0.0257 |  |
|  |  | **Total** |  | **140** | **0.4431** |  |  | **79** | **0.4202** |  |  | **61** | **0.4766** |  |  | **240** | **0.5129** |  |
| **Caucasian** | DRB1*03:01 | DQB1*02:01 |  | 14 | 0.0443 | 1.0000 |  | 9 | 0.0479 | 1.0000 |  | 5 | 0.0391 | 1.0000 |  | 15 | 0.0320 | 1.0000 |
|  | DRB1*11:04 | DQB1*03:01 |  | 11 | 0.0348 | 0.8199 |  | 2 | 0.0106 | 0.6179 |  | 9 | 0.0703 | 0.8792 |  | 8 | 0.0171 | 1.0000 |
|  | DRB1*15:01 | DQB1*06:02 |  | 8 | 0.0253 | 0.7921 |  | 5 | 0.0266 | 0.8269 |  | 3 | 0.0234 | 0.7398 |  | 15 | 0.0320 | 0.8779 |
|  | DRB1*13:01 | DQB1*06:03 |  | 7 | 0.0222 | 1.0000 |  | 4 | 0.0213 | 1.0000 |  | 3 | 0.0234 | 1.0000 |  | 6 | 0.0128 | 1.0000 |
|  | DRB1*11:01 | DQB1*03:01 |  | 5 | 0.0158 | 1.0000 |  | 4 | 0.0213 | 1.0000 |  | 1 | 0.0078 | 1.0000 |  | 2 | 0.0043 | 0.1130 |
|  | DRB1*04:01 | DQB1*03:02 |  | 2 | 0.0063 | 0.3190 |  | 1 | 0.0053 | 0.0433 |  | 1 | 0.0078 | 1.0000 |  | 3 | 0.0064 | 1.0000 |
|  | DRB1*04:02 | DQB1*03:02 |  | 2 | 0.0063 | 1.0000 |  | 1 | 0.0053 | 1.0000 |  | 1 | 0.0078 | 1.0000 |  | 10 | 0.0214 | 1.0000 |
|  | Other |  |  | 0 |  |  |  | 0 |  |  |  | 0 |  |  |  | 7 | 0.0151 |  |
|  |  | **Total** |  | **49** | **0.1551** |  |  | **26** | **0.1383** |  |  | **23** | **0.1797** |  |  | **66** | **0.1411** |  |
| **Caucasian** | DRB1*04:04 | DQB1*03:02 |  | 17 | 0.0538 | 1.0000 |  | 15 | 0.0798 | 1.0000 |  | 2 | 0.0156 | 1.0000 |  | 29 | 0.0620 | 0.9144 |
| **Shared** | DRB1*01:02 | DQB1*05:01 |  | 16 | 0.0506 | 1.0000 |  | 11 | 0.0585 | 1.0000 |  | 5 | 0.0391 | 1.0000 |  | 11 | 0.0235 | 1.0000 |
|  | DRB1*01:01 | DQB1*05:01 |  | 12 | 0.0380 | 1.0000 |  | 10 | 0.0532 | 1.0000 |  | 2 | 0.0156 | 1.0000 |  | 9 | 0.0192 | 1.0000 |
|  | DRB1*07:01 | DQB1*02:02 |  | 10 | 0.0316 | 0.9055 |  | 6 | 0.0319 | 0.8516 |  | 4 | 0.0313 | 1.0000 |  | 28 | 0.0598 | 1.0000 |
|  | DRB1*04:03 | DQB1*03:02 |  | 9 | 0.0285 | 0.7524 |  | 6 | 0.0319 | 0.7950 |  | 3 | 0.0234 | 0.6832 |  | 10 | 0.0214 | 1.0000 |
|  | DRB1*04:05 | DQB1*03:02 |  | 6 | 0.0190 | 0.8045 |  | 4 | 0.0213 | 0.7130 |  | 2 | 0.0156 | 1.0000 |  | 1 | 0.0022 | 1.0000 |
|  | DRB1*08:01 | DQB1*04:02 |  | 5 | 0.0158 | 1.0000 |  | 3 | 0.0160 | 1.0000 |  | 2 | 0.0156 | 1.0000 |  | 1 | 0.0022 | 1.0000 |
|  | DRB1*14:01 | DQB1*05:03 |  | 4 | 0.0127 | 1.0000 |  | 3 | 0.0160 | 1.0000 |  | 1 | 0.0078 | 1.0000 |  | 8 | 0.0171 | 1.0000 |
|  | DRB1*13:02 | DQB1*06:04 |  | 2 | 0.0063 | 1.0000 |  | 1 | 0.0053 | 1.0000 |  | 1 | 0.0078 | 1.0000 |  | 9 | 0.0192 | 0.8978 |
|  | DRB1*04:01 | DQB1*03:01 |  | 2 | 0.0063 | 0.4148 |  | 2 | 0.0106 | 0.6179 |  | ND |  |  |  | ND |  |  |
|  | DRB1*07:01 | DQB1*03:03 |  | 2 | 0.0063 | 1.0000 |  | 1 | 0.0053 | 1.0000 |  | 1 | 0.0078 | 1.0000 |  | 5 | 0.0107 | 0.4620 |
|  | DRB1*01:03 | DQB1*05:01 |  | 1 | 0.0032 | 1.0000 |  | ND |  |  |  | 1 | 0.0078 | 1.0000 |  | 3 | 0.0064 | 1.0000 |
|  | DRB1*04:03 | DQB1*03:01 |  | 1 | 0.0032 | -0.3755 |  | 1 | 0.0053 | 0.0174 |  | ND |  |  |  | ND |  |  |
|  | DRB1*04:08 | DQB1*03:01 |  | 1 | 0.0032 | 1.0000 |  | ND |  |  |  | 1 | 0.0078 | 1.0000 |  | 1 | 0.0022 | 1.0000 |
|  | Other |  |  | 0 |  |  |  | 0 |  |  |  | 0 |  |  |  | 14 | 0.0302 |  |
|  |  | **Total** |  | **88** | **0.2785** |  |  | **63** | **0.3351** |  |  | **25** | **0.1953** |  |  | **129** | **0.2761** |  |
| **African** | DRB1*15:03 | DQB1*06:02 |  | 4 | 0.0127 | 1.0000 |  | 2 | 0.0106 | 1.0000 |  | 2 | 0.0156 | 1.0000 |  | 1 | 0.0022 | 1.0000 |
|  | DRB1*08:04 | DQB1*03:01 |  | 3 | 0.0095 | 1.0000 |  | 1 | 0.0053 | 1.0000 |  | 2 | 0.0156 | 1.0000 |  | 2 | 0.0043 | 1.0000 |
|  | DRB1*13:02 | DQB1*06:09 |  | 3 | 0.0095 | 1.0000 |  | 2 | 0.0106 | 1.0000 |  | 1 | 0.0078 | 1.0000 |  | ND |  |  |
|  | DRB1*10:01 | DQB1*05:01 |  | 2 | 0.0063 | 1.0000 |  | ND |  |  |  | 2 | 0.0156 | 1.0000 |  | 5 | 0.0107 | 0.8211 |
|  | DRB1*03:02 | DQB1*04:02 |  | 1 | 0.0032 | 1.0000 |  | ND |  |  |  | 1 | 0.0078 | 1.0000 |  | 1 | 0.0022 | 1.0000 |
|  | DRB1*13:02 | DQB1*05:01 |  | 1 | 0.0032 | 0.0728 |  | ND |  |  |  | 1 | 0.0078 | 0.2707 |  | ND |  |  |
|  | Other |  |  | 0 |  |  |  | 0 |  |  |  | 0 |  |  |  | 7 | 0.0152 |  |
|  |  | **Total** |  | **14** | **0.0443** |  |  | **5** | **0.0266** |  |  | **9** | **0.0703** |  |  | **16** | **0.0346** |  |
| **Asian** | DRB1*15:02 | DQB1*06:01 |  | 4 | 0.0127 | 0.7968 |  | 3 | 0.0160 | 0.7446 |  | 1 | 0.0078 | 1.0000 |  | 5 | 0.0107 | 1.0000 |
|  | DRB1*11:02 | DQB1*03:01 |  | 1 | 0.0032 | 0.1222 |  | ND |  |  |  | 1 | 0.0078 | 0.1950 |  | 3 | 0.0064 | 0.6674 |
|  | DRB1*12:01 | DQB1*03:01 |  | 1 | 0.0032 | 1.0000 |  | 1 | 0.0053 | 1.0000 |  | ND |  |  |  | 1 | 0.0022 | 0.3348 |
|  | Other |  |  | 0 |  |  |  | 0 |  |  |  | 0 |  |  |  | 1 | 0.0022 |  |
|  |  | **Total** |  | **6** | **0.0190** |  |  | **4** | **0.0213** |  |  | **2** | **0.0156** |  |  | **10** | **0.0216** |  |
| **UNKOWN** | DRB1*11:02 | DQB1*03:19 |  | 3 | 0.0095 | 0.7452 |  | 1 | 0.0053 | 1.0000 |  | 2 | 0.0156 | 0.6587 |  | 1 | 0.0022 | 0.2419 |
|  | DRB1*13:04 | DQB1*03:19 |  | 3 | 0.0095 | 1.0000 |  | 2 | 0.0106 | 1.0000 |  | 1 | 0.0078 | 1.0000 |  | ND |  |  |
|  | DRB1*04:06 | DQB1*04:02 |  | 2 | 0.0063 | 1.0000 |  | 2 | 0.0106 | 1.0000 |  | ND |  |  |  | ND |  |  |
|  | DRB1*14:06 | DQB1*06:03 |  | 1 | 0.0032 | 0.0614 |  | ND |  |  |  | 1 | 0.0078 | 0.1607 |  | ND |  |  |
|  | DRB1*15:01 | DQB1*06:03 |  | 1 | 0.0032 | 0.0706 |  | ND |  |  |  | 1 | 0.0078 | 0.2131 |  | ND |  |  |
|  | DRB1*08:02 | DQB1*03:01 |  | 1 | 0.0032 | -0.9004 |  | ND |  |  |  | 1 | 0.0078 | -0.8289 |  | ND |  |  |
|  | DRB1*04:03 | DQB1*03:05 |  | 1 | 0.0032 | 1.0000 |  | ND |  |  |  | 1 | 0.0078 | 1.0000 |  | ND |  |  |
|  | DRB1*11:04 | DQB1*06:03 |  | 1 | 0.0032 | 0.0614 |  | ND |  |  |  | 1 | 0.0078 | 0.0960 |  | ND |  |  |
|  | DRB1*11:04 | DQB1*05:02 |  | 1 | 0.0032 | 0.4785 |  | 1 | 0.0053 | 0.4919 |  | ND |  |  |  | ND |  |  |
|  | DRB1*04:05 | DQB1*02:02 |  | 1 | 0.0032 | 0.1119 |  | 1 | 0.0053 | 0.1691 |  | ND |  |  |  | ND |  |  |
|  | DRB1*11:03 | DQB1*03:01 |  | 1 | 0.0032 | 1.0000 |  | 1 | 0.0053 | 1.0000 |  | ND |  |  |  | ND |  |  |
|  | DRB1*15:02 | DQB1*05:02 |  | 1 | 0.0032 | 0.4920 |  | 1 | 0.0053 | 0.4891 |  | ND |  |  |  | ND |  |  |
|  | DRB1*03:15 | DQB1*02:01 |  | 1 | 0.0032 | 1.0000 |  | 1 | 0.0053 | 1.0000 |  | ND |  |  |  | ND |  |  |
|  | DRB1*15:01 | DQB1*06:01 |  | 1 | 0.0032 | 0.1739 |  | 1 | 0.0053 | 0.2253 |  | ND |  |  |  | ND |  |  |
|  | Other |  |  | 0 |  |  |  | 0 |  |  |  | 0 |  |  |  | 6 | 0.0131 |  |
|  |  | **Total** |  | **19** | **0.0601** |  |  | **11** | **0.0585** |  |  | **8** | **0.0625** |  |  | **7** | **0.0153** |  |

| **Section 8. MHC conserved extended haplotypes in Mexican SSc patients and healthy controls.** | | | | | | | | | | | | | | | | | | | | |
| --- | --- | --- | --- | --- | --- | --- | --- | --- | --- | --- | --- | --- | --- | --- | --- | --- | --- | --- | --- | --- |
|  |  |  |  |  |  | **SSc (N=316)** | |  |  | **Limited (N=188)** | | |  | **Diffuse (N=128)** | | |  | **Controls (N=468)** | | |
|  | **C-B-DRB1-DQB1 haplotype** | | |  |  | **n** | **H.F.** | **D'** |  | **n** | **H.F.** | **D'** |  | **n** | **H.F.** | **D'** |  | **n** | **H.F.** | **D'** |
| **Amerindian** | C*04:01 | B*35:17 | DRB1*08:02 | DQB1*04:02 |  | 15 | 0.0474 | 0.7002 |  | 11 | 0.0585 | 0.6928 |  | 4 | 0.0312 | 0.7305 |  | 14 | 0.0299 | 0.7256 |
|  | C*04:01 | B*35:12 | DRB1*08:02 | DQB1*04:02 |  | 12 | 0.0379 | 0.4903 |  | 7 | 0.0372 | 0.3856 |  | 5 | 0.0390 | 0.3263 |  | 7 | 0.0150 | 0.3054 |
|  | C*07:02 | B*39:05 | DRB1*04:07 | DQB1*03:02 |  | 10 | 0.0316 | 0.2283 |  | 8 | 0.0425 | 0.7665 |  | 2 | 0.0156 | 1.0000 |  | 19 | 0.0406 | 0.5025 |
|  | C*01:02 | B*15:15 | DRB1*08:02 | DQB1*04:02 |  | 9 | 0.0284 | 0.8938 |  | 6 | 0.0319 | 0.2320 |  | 3 | 0.0234 | 1.0000 |  | 8 | 0.0171 | 0.5251 |
|  | C*07:02 | B*39:06 | DRB1*04:07 | DQB1*03:02 |  | 6 | 0.0189 | 0.2238 |  | 5 | 0.0265 | 0.2215 |  | 1 | 0.0078 | 0.2381 |  | 4 | 0.0086 | 0.0394 |
|  | C*04:01 | B*35:12 | DRB1*04:07 | DQB1*03:02 |  | 5 | 0.0158 | 0.1319 |  | 3 | 0.0159 | 0.1242 |  | 2 | 0.0156 | 0.2857 |  | 2 | 0.0043 | 0.0133 |
|  | C*07:02 | B*39:06 | DRB1*14:06 | DQB1*03:01 |  | 4 | 0.0126 | 0.3474 |  | 2 | 0.0106 | 0.3208 |  | 2 | 0.0156 | 1.0000 |  | 16 | 0.0342 | 0.5482 |
|  | C*08:01 | B*48:01 | DRB1*08:02 | DQB1*04:02 |  | 3 | 0.0094 | 0.3629 |  | 1 | 0.0053 | 1.0000 |  | 2 | 0.0156 | 0.5509 |  | 8 | 0.0171 | 1.0000 |
|  | C*04:01 | B*35:17 | DRB1*04:07 | DQB1*03:02 |  | 3 | 0.0094 | 0.0468 |  | 2 | 0.0106 | -0.4198 |  | 1 | 0.0078 | 0.0857 |  | ND |  |  |
|  | C*01:02 | B*15:30 | DRB1*08:02 | DQB1*04:02 |  | 2 | 0.0063 | 0.2355 |  | ND |  |  |  | 2 | 0.0156 | 0.3263 |  | 4 | 0.0086 | 0.3826 |
|  | C*03:05 | B*40:02 | DRB1*04:07 | DQB1*03:02 |  | 2 | 0.0063 | 0.6142 |  | 1 | 0.0053 | 1.0000 |  | 1 | 0.0078 | 1.0000 |  | 4 | 0.0086 | 0.3234 |
|  | C*07:02 | B*39:05 | DRB1*08:02 | DQB1*04:02 |  | 2 | 0.0063 | -0.6127 |  | 2 | 0.0106 | -0.4629 |  | ND |  |  |  | 5 | 0.0107 | -0.2267 |
|  |  |  |  | Other |  | 0 |  |  |  | 0 |  |  |  | 0 |  |  |  | 33 |  |  |
|  |  |  |  | **Total** |  | **73** | **0.2303** |  |  | **48** | **0.2549** |  |  | **25** | **0.1950** |  |  | **124** | **0.2649** |  |
| **Caucasian** | C*08:02 | B*14:02 | DRB1*01:02 | DQB1*05:01 |  | 8 | 0.0253 | 0.7367 |  | 5 | 0.0265 | 0.7345 |  | 3 | 0.0234 | 0.7398 |  | 5 | 0.0107 | 0.4414 |
|  | C*07:01 | B*08:01 | DRB1*03:01 | DQB1*02:01 |  | 7 | 0.0221 | 0.6195 |  | 5 | 0.0265 | 0.0266 |  | 2 | 0.0156 | 0.3756 |  | 2 | 0.0043 | 0.6556 |
|  | C*12:03 | B*18:01 | DRB1*11:04 | DQB1*03:01 |  | 5 | 0.0158 | 0.8273 |  | ND |  |  |  | 5 | 0.0390 | 1.0000 |  | ND |  |  |
|  | C*05:01 | B*18:01 | DRB1*03:01 | DQB1*02:01 |  | 2 | 0.0063 | 0.4768 |  | 1 | 0.0053 | 0.2998 |  | 1 | 0.0078 | 1.0000 |  | 3 | 0.0064 | 0.5868 |
|  | C*08:02 | B*14:01 | DRB1*07:01 | DQB1*02:02 |  | 2 | 0.0063 | 0.6558 |  | 2 | 0.0106 | 1.0000 |  | ND |  |  |  | 3 | 0.0064 | 0.7341 |
|  | C*04:01 | B*35:01 | DRB1*04:04 | DQB1*03:02 |  | 2 | 0.0063 | 0.0675 |  | 2 | 0.0106 | 0.1548 |  | ND |  |  |  | 3 | 0.0064 | 0.1472 |
|  | C*04:01 | B*35:01 | DRB1*01:01 | DQB1*05:01 |  | 2 | 0.0063 | 0.1193 |  | 2 | 0.0106 | 0.0612 |  | ND |  |  |  | 1 | 0.0021 | 0.7265 |
|  |  |  |  | Other |  | 0 |  |  |  | 0 |  |  |  | 0 |  |  |  | 20 |  |  |
|  |  |  |  | **Total** |  | **28** | **0.0884** |  |  | **17** | **0.0901** |  |  | **11** | **0.0858** |  |  | **36** | **0.0771** |  |
| **Asian** | C*12:02 | B*52:01 | DRB1*15:02 | DQB1*06:01 |  | 2 | 0.0063 | 1.0000 |  | 1 | 0.0053 | 1.0000 |  | 1 | 0.0078 | 1.0000 |  | ND |  |  |
|  |  |  |  | Other |  | 0 |  |  |  | 0 |  |  |  | 0 |  |  |  | 2 |  |  |
|  |  |  |  | **Total** |  | **2** | **0.0063** |  |  | **1** | **0.0053** |  |  | **1** | **0.0078** |  |  | **2** | **0.0043** |  |
| **Unknown** | C*04:01 | B*35:01 | DRB1*08:02 | DQB1*04:02 |  | 5 | 0.0158 | 0.0256 |  | ND |  |  |  | 5 | 0.0390 | 0.3263 |  | 3 | 0.0064 | 0.0121 |
|  | C*03:06 | B*40:02 | DRB1*08:02 | DQB1*04:02 |  | 4 | 0.0126 | 0.3629 |  | ND |  |  |  | 4 | 0.0312 | 0.1916 |  | ND |  |  |
|  | C*01:02 | B*15:01 | DRB1*08:02 | DQB1*04:02 |  | 3 | 0.0094 | 0.4903 |  | 3 | 0.0159 | 0.5085 |  | ND |  |  |  | 3 | 0.0064 | 0.2944 |
|  | C*06:02 | B*45:01 | DRB1*01:01 | DQB1*05:01 |  | 3 | 0.0094 | 0.4803 |  | 1 | 0.0053 | 1.0000 |  | 2 | 0.0156 | 0.4880 |  | ND |  |  |
|  | C*04:01 | B*53:01 | DRB1*01:02 | DQB1*05:01 |  | 3 | 0.0094 | 0.2978 |  | 2 | 0.0106 | 0.2034 |  | 1 | 0.0078 | 0.4921 |  | ND |  |  |
|  | C*03:03 | B*52:01 | DRB1*04:11 | DQB1*03:02 |  | 3 | 0.0094 | 0.5923 |  | 2 | 0.0106 | 0.6613 |  | 1 | 0.0078 | 0.4880 |  | 1 | 0.0021 | 0.7201 |
|  | C*07:01 | B*49:01 | DRB1*04:05 | DQB1*03:02 |  | 3 | 0.0094 | 0.4903 |  | 1 | 0.0053 | -1.0000 |  | 2 | 0.0156 | 1.0000 |  | ND |  |  |
|  | C*08:01 | B*51:01 | DRB1*04:11 | DQB1*04:02 |  | 2 | 0.0063 | 0.2355 |  | 1 | 0.0053 | 0.4891 |  | 1 | 0.0078 | 0.3263 |  | ND |  |  |
|  | C*01:02 | B*15:30 | DRB1*04:03 | DQB1*03:02 |  | 2 | 0.0063 | 0.3804 |  | 2 | 0.0106 | 0.3114 |  | ND |  |  |  | 1 | 0.0021 | 1.0000 |
|  | C*04:01 | B*35:12 | DRB1*04:03 | DQB1*03:02 |  | 2 | 0.0063 | 0.1459 |  | 1 | 0.0053 | 0.1000 |  | 1 | 0.0078 | 0.2889 |  | ND |  |  |
|  | C*15:02 | B*40:02 | DRB1*04:07 | DQB1*03:02 |  | 2 | 0.0063 | 0.2283 |  | 2 | 0.0106 | 0.3188 |  | ND |  |  |  | ND |  |  |
|  | C*01:02 | B*27:05 | DRB1*01:01 | DQB1*05:01 |  | 2 | 0.0063 | 1.0000 |  | 2 | 0.0106 | 1.0000 |  | ND |  |  |  | ND |  |  |
|  | C*04:01 | B*35:02 | DRB1*11:04 | DQB1*03:01 |  | 2 | 0.0063 | 0.6546 |  | 1 | 0.0053 | 1.0000 |  | 1 | 0.0078 | 0.4622 |  | ND |  |  |
|  | C*07:02 | B*39:06 | DRB1*04:04 | DQB1*03:02 |  | 2 | 0.0063 | 0.0644 |  | 2 | 0.0106 | 0.0582 |  | ND |  |  |  | 2 | 0.0064 | 0.0129 |
|  | C*14:02 | B*51:01 | DRB1*08:01 | DQB1*04:02 |  | 2 | 0.0063 | 0.6613 |  | 1 | 0.0053 | 0.4919 |  | 1 | 0.0078 | 1.0000 |  | ND |  |  |
|  | C*03:04 | B*40:02 | DRB1*04:07 | DQB1*03:02 |  | 2 | 0.0063 | 0.4212 |  | 1 | 0.0053 | 0.2215 |  | 1 | 0.0078 | 0.4286 |  | ND |  |  |
|  | C*08:01 | B*51:02 | DRB1*08:02 | DQB1*04:02 |  | 2 | 0.0063 | 0.5753 |  | ND |  |  |  | 2 | 0.0156 | 0.5509 |  | 1 | 0.0021 | 1.0000 |
|  | C*06:02 | B*45:01 | DRB1*01:02 | DQB1*05:01 |  | 2 | 0.0063 | 0.2100 |  | ND |  |  |  | 2 | 0.0156 | 0.3062 |  | ND |  |  |
|  |  |  |  | Other |  | 0 |  |  |  | 0 |  |  |  | 0 |  |  |  | 70 |  |  |
|  |  |  |  | **Total** |  | **49** | **0.1541** |  |  | **25** | **0.1325** |  |  | **24** | **0.1872** |  |  | **84** | **0.1868** |  |
